# A Model of *In vitro* Plasticity at the Parallel Fiber—Molecular Layer Interneuron Synapses

**DOI:** 10.3389/fncom.2015.00150

**Published:** 2015-12-24

**Authors:** William Lennon, Tadashi Yamazaki, Robert Hecht-Nielsen

**Affiliations:** ^1^Department of Electrical and Computer Engineering, University of CaliforniaSan Diego, La Jolla, CA, USA; ^2^Graduate School of Informatics and Engineering, The University of Electro-CommunicationsChofu, Japan

**Keywords:** molecular layer interneurons, parallel fibers, plasticity, cerebellum, gated steepest descent

## Abstract

Theoretical and computational models of the cerebellum typically focus on the role of parallel fiber (PF)—Purkinje cell (PKJ) synapses for learned behavior, but few emphasize the role of the molecular layer interneurons (MLIs)—the stellate and basket cells. A number of recent experimental results suggest the role of MLIs is more important than previous models put forth. We investigate learning at PF—MLI synapses and propose a mathematical model to describe plasticity at this synapse. We perform computer simulations with this form of learning using a spiking neuron model of the MLI and show that it reproduces six *in vitro* experimental results in addition to simulating four novel protocols. Further, we show how this plasticity model can predict the results of other experimental protocols that are not simulated. Finally, we hypothesize what the biological mechanisms are for changes in synaptic efficacy that embody the phenomenological model proposed here.

## Introduction

The parallel fiber (PF)—Purkinje cell (PKJ) excitatory synapse has historically been considered the locus of learning and memory in the cerebellar cortex, driven by climbing fiber (CF) inputs (Grossberg, 1969; Marr, 1969; Albus, 1971; Ito et al., 2014). While the number of PF-PKJ synapses formed and capacity for information storage is massive (Brunel et al., 2004; Ito, 2006), excitatory inputs to PKJs alone do not account for decreases in PKJ activity observed during behavior (Miyashita and Nagao, 1984; Jirenhed et al., 2007) since PKJs fire spontaneously in absence of excitatory inputs (Häusser and Clark, 1997; Cerminara and Rawson, 2004). The molecular layer interneurons (MLI)—stellate and basket cells—also receive PF inputs and provide feedforward inhibitory inputs to the PKJs in addition to the recurrent inhibitory inputs they form with other MLIs (Eccles et al., 1967; Palay and Chan-Palay, 1974). Learned changes to PF-MLI and MLI-PKJ synapses are postulated to increase the information capacity of the MLI-PKJ network and richness of PKJ output dynamics (Albus, 1971; Dean et al., 2010), however relatively little is known about plasticity at these synapses.

There is mounting experimental evidence that MLIs play an important role in cerebellar function. Genetically modified mice lacking PKJ gamma-aminobutyric acid A (GABA_A_) receptors exhibit significant motor learning deficits (Wulff et al., 2009), suggesting a significant functional role for MLI feedforward inhibition in motor learning. Further, using optogenetics to selectively modulate the firing rates of MLIs via photostimulation elicits movement and controls movement kinematics in awake mice (Heiney et al., 2014). Thus, orchestrated MLI activity is functionally capable of controlling the gain and timing of movement components. Understanding the learned changes in MLI feedforward inhibition onto PKJs is crucial to understanding the learned output of the cerebellar cortex.

Previous *in vivo* studies showing CF driven changes to the PF-MLI receptive fields (RF) led to the hypothesis that concomitant PF and CF activation strengthens PF-MLI synapses and PF stimulation alone weakens them (Jörntell and Ekerot, 2002, 2003, 2011). Thus, this form of learning is said to be complementary and synergistic to PF-PKJ learning (Gao et al., 2012), but the mechanisms governing these receptive field changes are not understood. There is, however, a diverse body of *in vitro* experimental evidence where the mechanisms governing plasticity have been directly investigated and these results suggest that bidirectional changes in synaptic efficacy can occur in absence of CF activity (Liu and Cull-Candy, 2000; Rancillac and Crépel, 2004; Smith and Otis, 2005; Sun and June Liu, 2007; Kelly et al., 2009).

In this study, we propose a mathematical model of learning at PF-MLI synapses that is consistent with *in vitro* experimental findings. We choose to model plasticity at a single synapse based on the *in vitro* evidence since there is a larger and more diverse body of data that informs the model of the mechanisms involved. The model is developed with the *in vivo* evidence in mind and possible extensions to the model are proposed that could bridge the gap between synaptic and receptive field changes. It is worth cautioning that *in vitro* results may not hold under *in vivo* conditions and the model should be considered carefully until validated *in vivo*. We perform computer simulations using this model of plasticity and spiking neuron models to reproduce six *in vitro* experimental results and simulate four novel protocols. Finally, we hypothesize what the biological mechanisms underlying this model are and interpret the experimental results in terms of the model and mechanisms.

## Methods

### Neuron model

The MLI neuron model is similar to the neuron model we used in our previous simulations (Lennon et al., 2014) except that it excludes inhibitory synaptic conductances and includes excitatory synaptic conductances described below. Briefly, the MLI is modeled as a conductance-based leaky integrate-and-fire neuron model (Gerstner and Kistler, 2002) with α-Amino-3-hydroxy-5-methyl-4-isoxazolepropionic acid (AMPA) and N-Methyl-D-aspartic acid (NMDA) conductances and an intrinsic depolarizing current which is drawn from a gamma distribution, *I*_*spont*_(*t*) ~ Γ(κ, β) (in units nA), that causes the neuron to fire spontaneously. When the membrane potential of the neuron reaches *V*_*threshold*_, the neuron emits a spike and an after-hyperpolarization conductance, *g*_*ahp*_(*t*), is activated. *g*_*ahp*_(*t*) is modeled according to Equation (2), where *t*_*spiked*_ is the time the neuron last spiked and τ_*ahp*_ is the conductance time constant. Actual parameters used are summarized in Table 1.

(1) $$\begin{array}{l} C\frac{dV}{dt}=-g_{leak}(V(t)-E_{leak})-g_{ahp}(t)(V(t)-E_{ahp})-(g_{AMPA}(t)\\ \;+\;g_{NMDA}(t))(V(t)-E_{exc})+I_{spont}(t) \end{array}$$

(2) $$g_{ahp}(t-t_{spiked})=\exp\left(\frac{-(t-t_{spiked})}{\tau_{ahp}}\right)$$

**Table 1:** **Summary of simulation parameters**.

**MLI neuron parameters**	**Value**
*V*_*threshold*_ (mV)	−53.0
*C* (pF)	14.6
ḡ_*leak*_ (nS)	1.6
*E*_*leak*_ (mV)	−68.0
ḡ_*AMPA*_ (nS)	3.0
*E*_*exc*_ (mV)	0.0
τ_*fast*_ (ms)	0.8
τ_*slow*_ (ms)	18.0
α_*fast*_	0.8
α_*slow*_	0.2
ḡ_*NMDA*_ (nS)	1.0
τ_*rise*_ (ms)	3.0
τ_*decay*_ (ms)	40.0
τ_*n*_ (ms)	10.0
ḡ_*AHP*_ (nS)	50.0
*E*_*AHP*_ (mV)	−82.0
τ_*AHP*_ (msec)	2.5
κ	3.966333
β	0.006653

*MLI biophysical parameters (Midtgaard, 1992; Häusser and Clark, 1997; Carter and Regehr, 2002; Lachamp et al., 2009) AMPA receptor parameters (Carter and Regehr, 2002; Satake et al., 2012); NMDA receptor parameters (Gabbiani et al., 1994); τ_n_ derived by hand to match (Carter and Regehr, 2002); κ, β from (Lennon et al., 2014)*.

Granule cells were not modeled directly and instead we simulated the arrival of PF spikes to PF-MLI synapses according to Poisson statistics with variable rate λ(*t*) which is controlled during simulations.

### Synaptic conductances

Model PF-MLI synapses contain both AMPA and NMDA receptor conductances. Total AMPA synaptic conductances are computed according to Equation (3), where ḡ_*syn*_ is the maximal synaptic conductance, *w*_*i*_ is the synaptic weight, α(*t*) is the synaptic conductance kinetics function, and δ_*i*_(*t*) is a Dirac delta function for the *i*th synapse onto the target neuron, indicating whether the pre-synaptic neuron has spiked at time t. We use the terms “synaptic weight,” “synaptic efficacy,” and “synaptic strength” interchangeably. PF-MLI AMPAR conductance rise times are modeled as instantaneous increases whereas decay times are modeled as double exponentials to approximately fit the prolonged conductances at these synapses (Carter and Regehr, 2000; Equation 4).

(3) $$g_{AMPA}(t)={\bar{g}}_{AMPA}\sum_{i}w_{i}\int_{-\infty }^{t}\alpha_{AMPA}(t-s)\delta_{i}(s)ds$$

(4) $$\alpha_{AMPA}\left(t\right)=\;\alpha_{fast}\exp\left(-\frac{t}{\tau_{fast}}\right)+\alpha_{slow}\exp\left(-\frac{t}{\tau_{slow}}\right)$$

PF-MLI synaptic weights are modeled with a fixed minimum value, *w*_0_ ∈ (0, 1), and a variable component, ${\hat{w}}_{i}\in \left[0,1\right]$ that changes according to the weight update equation described in the next section. Table 2 shows actual value of *w*_0_ used. Equation (5) ensures the effective synaptic weight, *w*_*i*_, lies within the range [*w*_0_, 1].

(5) $$w_{i}=w_{0}+\;(1-w_{0}){\hat{w}}_{i}$$

**Table 2:** **Learning rule parameters**.

**Neuron trace and weight update parameters**	**MLI**	**PF**
τ_ψ_(ms)	60.0	10.0
ν_ψ_(ms)	15.0	2.0
*f*_max_(Hz)	150	300
η	0.001	
*w*_0_	0.2	

Voltage sensitive NMDAR conductances are modeled in accordance with Equations (6)–(8) which roughly capture the neurotransmitter availability in the synaptic cleft and the opening and closing kinetics of NMDA receptors, respectively. Where ρ = (3.57mM)^−1^, ${\left[Mg^{2+}\right]}_{o}=1.2\text{mM}$ is the extracellular magnesium concentration, and σ = (−0.062mV)^−1^ (McCormick et al., 1993; Gabbiani et al., 1994). The logarithm of *n*(*t*) in Equation (7) is used for numerical stability.

(6) $$n\left(t\right)=\int_{-\infty }^{t}\exp\left(-\frac{t-s}{\tau_{n}}\right)\delta \left(s\right)ds$$

(7) $$\frac{dR}{dt}=\frac{\log\left(n\left(t\right)+1\right)\left(1-R\right)}{\tau_{rise}}-R/\tau_{decay}$$

(8) $$g_{NMDA}\left(t\right)=\;{\bar{g}}_{NMDA}R{\left(1+\rho {\left[{\text{Mg}}^{\text{2+}}\right]}_{\text{o}}{\text{e}}^{\sigma \text{V}}\right)}^{-1}$$

### Neuron traces

A trace of the neuron spiking activity is calculated every time step of the simulation and used to compute a smooth measure of the instantaneous neuron firing rate that is normalized using a neuron specific maximum firing rate, *f*_*max*_ (Table 2). If the actual firing rate of the neuron exceeded *f*_*max*_, the trace is truncated to *f*_*max*_. This results in a unitless measure of the neuron firing activity bounded by zero and one, i.e., ${\bar{x}}_{i}\left(t\right)\in \left[0,1\right]$. Throughout the paper we refer to this as the neuron “activity trace” or “firing trace.” In several figures we plot the non-normalized “firing rate trace” (in units Hz) as well.

(9) $$\psi (t):=\frac{e^{\frac{-t}{\tau_{\psi }}}-e^{\frac{-t}{\nu_{\psi }}}}{\tau_{\psi }-\nu_{\psi }}$$

(10) $${\bar{x}}_{i}\left(t\right)=\frac{1}{f_{max}}\int_{-\infty }^{\text{t}}\psi \left(t-s\right)\delta_{i}\left(s\right)ds$$

### Synapse learning rule

Synapses are updated according to the *gated steepest descent* learning rule (Chen, 2007). The weight update is correlative based on the activity of the pre-synaptic neuron, ${\bar{\text{PF}}}_{\text{i}}\left(t\right)$, and the difference between the post-synaptic activity, ${\bar{\text{MLI}}}_{\text{j}}\left(t\right)$, and a measure of the synaptic strength, *w*_*i, j*_ · η is the learning rate parameter, and γ is a free parameter that is adjusted during certain experiments, but is otherwise set to one. A biological interpretation of the learning rule can be found in the Discussion.

(11) $$\frac{d{\hat{w}}_{i,j}}{dt}=\eta {\bar{\text{PF}}}_{i}\left(t\right)[{\bar{\text{MLI}}}_{\text{j}}\left(t\right)-\gamma {\hat{w}}_{i,j}]$$

### Software and data analysis

Simulations were performed in Python using BRIAN simulator—a spiking neural network framework (Goodman and Brette, 2009). All simulations are performed with a time step of 0.25 ms using Euler's method for integration of differential equations to ensure numerical stability. Data was analyzed and plotted using BRIAN Simulator, SciPy, Numpy, Matplotlib, Seaborn, and homemade software written in Python. Action potentials are drawn in plots by inserting a value of 0 mV in recordings of neuron model membrane potentials immediately after the model neuron reaches threshold. The source code for all experiments will be made freely available online upon publication.

## Results

### Model of synaptic plasticity

Plasticity at the PF-MLI synapses involves several mechanisms that produce both pre- and post-synaptic changes (Liu and Cull-Candy, 2000; Rancillac and Crépel, 2004; Smith and Otis, 2005; Soler-Llavina and Sabatini, 2006; Sun and June Liu, 2007; Bender et al., 2009; Kelly et al., 2009). Here, we present a *phenomenological* model of PF-MLI plasticity that is a function of PF and MLI activity and a measure of the synaptic efficacy, *w*. While we mainly focus on evidence for post-synaptic plasticity, it is expected that this model also accounts for pre-synaptic changes in synaptic efficacy in physiologically realistic conditions. Post-synaptic plasticity involves changes in the AMPA receptor phenotype composition (Liu and Cull-Candy, 2000, 2002; Liu and Savtchouk, 2012). Both LTP and LTD are observed and dependent on post-synaptic calcium signaling (Liu and Cull-Candy, 2000; Rancillac and Crépel, 2004; Smith and Otis, 2005).

We hypothesize that activity-dependent post-synaptic Ca^2+^ transients induce changes in post-synaptic plasticity. These transients can induce both LTD and LTP, dependent on a dynamic cytosolic Ca^2+^ threshold. We roughly capture the effects of activity dependent calcium transients as unit-less traces of the pre- ($\bar{\text{PF}}\left(t\right)$) and post-synaptic activities ($\bar{\text{MLI}}\left(t\right)$), and the dynamic threshold as a variable synaptic strength, *w*, multiplied by a variable scaling factor γ. For the purpose of these simulations, γ = 1 unless otherwise stated. η serves as a learning rate and is typically small, η = 0.001. This learning rule is also known as *gated steepest descent* (Chen, 2007) and is similar to the BCM learning rule (Bienenstock et al., 1982) and mechanistic models of calcium-dependent synaptic plasticity (Shouval et al., 2002).

(12) $$\frac{dw}{dt}=\eta \bar{\text{PF}}\left(t\right)[\bar{\text{MLI}}\left(t\right)-\gamma w]$$

Due to *w* serving as a dynamic threshold for plasticity, this learning rule exhibits LTP when $\bar{\text{PF}}\left(t\right)>0$ and $\bar{\text{MLI}}\left(t\right)>\gamma w$, and LTD when $\bar{\text{PF}}\left(t\right)>0$ and $\bar{\text{MLI}}\left(t\right)<\gamma w$ and is self-stabilizing so that synaptic weights do not “blow up.” The effect of this learning rule can be seen as *w* “chasing” the value of $\bar{\text{MLI}}\left(t\right)$, when the pre-synaptic activity is non-zero, i.e., $\bar{\text{PF}}\left(t\right)>0$, and the pre-synaptic activity serves as a dynamic learning rate. The learning rule is Hebbian in the sense that it is the sum of a correlative term, $\bar{\text{PF}}\left(t\right)\bar{\text{MLI}}\left(t\right)$, and a weight decay term, $-\gamma \bar{\text{PF}}\left(t\right)w$, which can be seen by multiplying the pre-synaptic activity term through. The model of synaptic efficacy implemented for the simulations described next has a fixed component to simulate a minimal synaptic efficacy and a variable component that is governed by Equation (12).

### Simulation of *in vitro* experiments

In this section, we present the results of computer simulations implementing this learning rule at PF-MLI synapses. The simulations consist of a single MLI spontaneously firing at about 30 Hz (simulating isolation from all inhibitory synaptic currents) with either a single PF or a bundle of 8 PFs forming synapses onto the MLI. Input spikes from PFs are modeled according to Poisson statistics with a variable rate which is controlled during the simulation. PF spikes produce both AMPA and NMDA conductances (described in Methods) in the MLI. In simulations I–IV, we simulate novel protocols to demonstrate the variety of synaptic weight changes depending on pre- and post-synaptic activities. In these simulations the simulated MLI receives only one PF input. In simulations V–X, we attempt to replicate the plasticity inducing protocols from a number of *in vitro* experiments and show that the simulations reproduce similar changes in synaptic efficacy. Table 3 summarizes the simulation protocols and results.

**Table 3:** **Summary of simulations**.

**Simulation**	**Simulation protocol**	**Result**	**Figure(s)**
I	Single PF bursts at 100 Hz, isolated MLI fires spontaneously at baseline. *n* = 10	LTP	1, 2
II	Single PF fires continuously at 10 Hz with a depolarizing current injected into an isolated MLI. *n* = 10	LTP	2, S1
III	Single PF fires continuously at 10 Hz with a hyperpolarizing current injected into an isolated MLI. *n* = 10	LTD	2, S2
IV	Single PF fires continuously at 2 Hz, isolated MLI fires spontaneously at baseline. *n* = 10	—	2, S3
V	A bundle of 8 PFs fires at 50 Hz while the target MLI is voltage clamped to −60 mV.	LTD	3, 5
VI	A bundle of 8 PFs fires 100 Hz bursts while the target MLI is current clamped to −80 mV.	LTP	4, 5
VII	A bundle of 8 PFs fires at 1 Hz while the target MLI is current clamped to −80 mV.	LTD	5, S4
VIII	A bundle of 8 PFs fires at 2 Hz while the target MLI is injected with a depolarizing current.	LTP	5, S5
IX	A bundle of 8 PFs fires at 1 Hz while the MLI fires spontaneously and the weight update parameter γ is increased to γ = 1.5.	LTD	5,S6
X	A bundle of 8 PFs fires at 1 Hz while the MLI fires spontaneously and the weight update parameter γ is decreased to γ = 0.5.	LTP	5, S7

#### Simulation I: high-frequency PF bursts induce LTP in spontaneously firing MLIs

**Figure 1:**
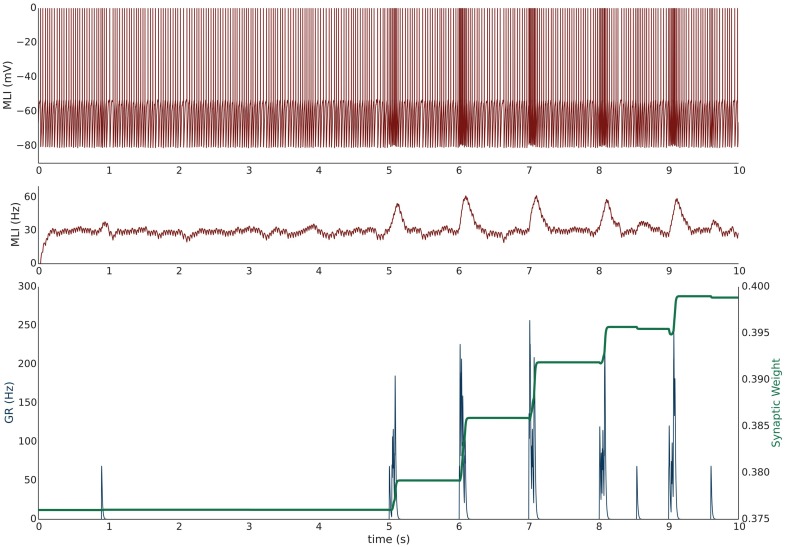
**Simulation I: PF-driven long term potentiation**. An example of one simulation run with an isolated MLI firing spontaneously (red traces; top panel: membrane potential; middle panel: firing rate trace) which receives one PF input (blue trace = the activity trace of a granule cell (GR) providing PF input). Only the first 10 s of the simulation are shown. The value of the synaptic strength, w, is shown in green in the lower panel and begins near its equilibrium value. During the first 5 s both PF and MLI firing at baseline at about 0.33 and 30 Hz, respectively. After 5 s, the PF fires 100 Hz bursts for 100 ms every 1 s. Starting at 5 s, each 1 s interval is considered a trial. The increased PF firing causes the MLI to increase its firing rate; at the same time, the synaptic weight, w, increases to compensate for the difference between the normalized MLI firing rate and the current value of *w*.

Simulation I investigates the effects of PF burst stimulation on the firing rate of a spontaneously firing isolated MLI and the consequent changes to synaptic efficacy. The simulation begins with a 5 s baseline period where the MLI and PF fire spontaneously at their baseline rates of 30 and 0.33 Hz respectively (Figure 1). After 5 s, the PF is then stimulated to fire approximately 100 Hz bursts (according to Poisson statistics) for 100 ms every 1 s. Each 1 s period starting after the baseline period constitutes one trial and 60 trials are simulated for a total simulation time of 65 s. The simulation is repeated independently 10 times (*n* = 10); Figure 1 shows the first 10 s of an example of one simulation. A recording of the MLI voltage is shown in the top panel (red) and a non-normalized firing rate trace is shown in the middle panel below (red). This firing rate trace is divided by a maximum firing rate parameter to normalize its value between [0,1] yielding $\bar{\text{MLI}}\left(t\right)$ which is used in the weight update equation (described in Methods). A non-normalized trace of the PF firing rate is shown in the bottom panel (blue). The value of the synaptic strength, *w*, starts near its equilibrium value (where $\hat{w}\approx \bar{\text{MLI}}\left(t\right)$ when PF stimulation is low but non-zero) and is plotted in the bottom panel (green). PF bursts increase the MLI firing rate above its baseline value. As the synaptic strength increases, we would expect a corresponding increase in the peak MLI firing rate during PF activation across trials. This is not always reflected in the first 10 s of the example simulation (e.g., Figure 1) since the PF burst is modeled as a Poisson spike train where the number and frequency of spikes varies slightly by trial.

**Figure 2:**
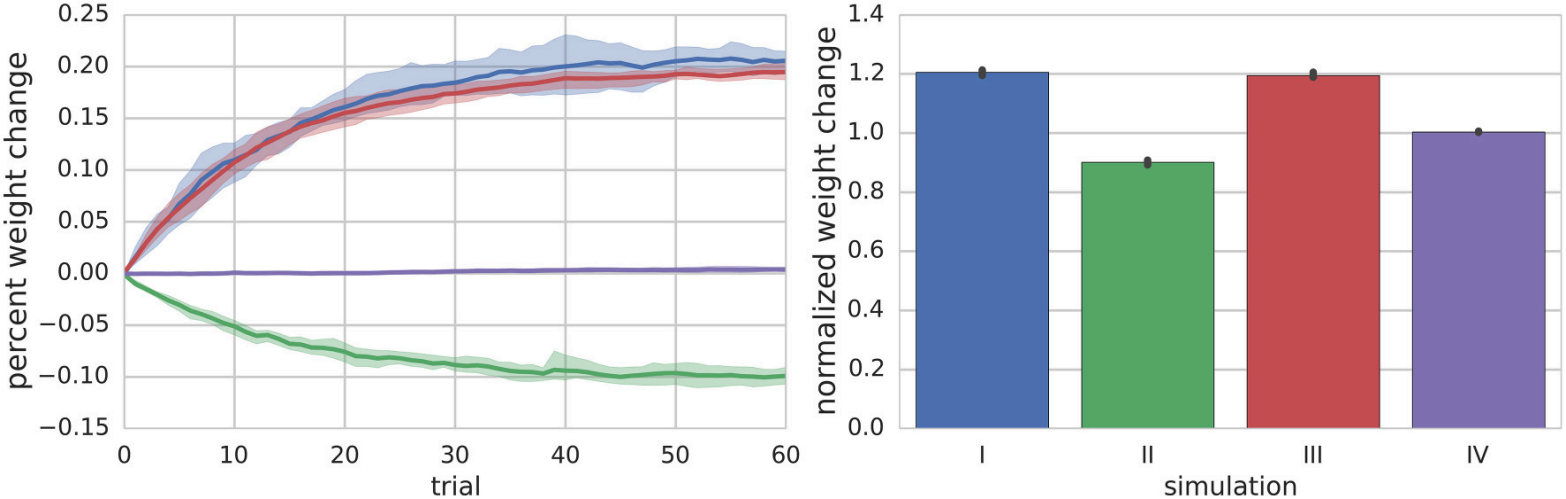
**Summary of Simulations I–IV**. The left panel shows the mean (solid lines) and range (shaded regions) percent change of synapse weight from starting values at the end of each trial across all simulated neurons (*n* = 10) for a particular simulation. For simulation I where the PF is stimulated to fire 100 Hz bursts for 100 ms every 1 s (i.e., 1 s trials), the synapse weights reach an equilibrium value of about 20% greater than starting values. The right panel shows the final mean (bar height) and range (black error bar) of weight values normalized to their starting values for each simulation.

During periods where PF activity is non-zero *and* where the normalized MLI firing activity (not shown) is greater than the variable weight component, $\hat{w}$, the synapse strengthens. The PF activity serves two functions: first to increase the MLI firing rate, and second to *gate* plasticity. The trajectory of the synaptic weight across trials and averaged over independent simulations is shown in Figure 2. One important feature of this learning rule is that the synaptic weight asymptotes instead of “blowing up” and is thus stable compared to a purely Hebbian rule. The asymptote is the result of the synaptic weight catching up to the MLI activity—as it gets closer, the change in synaptic efficacy is smaller.

#### Simulation II: continuous 10 Hz PF firing with paired MLI hyperpolarizing current injection induces LTD

Simulation II investigates the effects of a continuous 10 Hz stimulation of the PF with a paired hyperpolarizing current injected into the MLI to reduce its firing rate to approximately 10 Hz. The simulation begins with a 2.5 s baseline period where both PF and MLI fire spontaneously. Starting at 2.5 s, a hyperpolarizing current is injected into the MLI to reduce its firing rate to 10 Hz. Beginning at 5 s, the PF is continuously stimulated to fire at approximately 10 Hz (according to Poisson statistics) for the remainder of the simulation, 60 s (Supplementary Figure 1, analogous to Figure 1). Thus, the total simulation time is 65 s and we again refer to the 1 s periods beginning at 5 s as trials. During this period, where non-zero PF activity gates plasticity, the synaptic weight decreases since the MLI activity is below the synaptic weight value (Figure 2).

#### Simulation III: continuous 10 Hz PF firing with paired MLI depolarizing current injection induces LTP

Simulation III is identical to Simulation II except that a depolarizing current is injected into the MLI. Beginning at 2.5 s the MLI is injected with a constant depolarizing current sufficient to increase the MLI firing rate to approximately 40 Hz. Beginning at 5 s, the PF is continuously stimulated to fire at approximately 10 Hz (according to Poisson statistics) for the remainder of the simulation, 60 s (Supplementary Figure 2). Because the PF firing rate is non-zero and the MLI firing rate increases above baseline, the synaptic weight increases (Figure 2).

#### Simulation IV: low-frequency PF stimulation with spontaneous MLI firing results in unremarkable changes in synaptic efficacy

Simulation IV investigates the effect of a constant low frequency stimulation of the PF (2 Hz) while the MLI fires spontaneously at its baseline rate of 30 Hz. Similar to simulation I, a 5 s baseline period is simulated where the PF and MLI fire at 0.33 Hz and 30 Hz, respectively. After 5 s, for another 60 s, the PF fires at 2 Hz (according to Poisson statistics). Since this PF firing rate does not sufficiently change the MLI firing rate and the weight value begins near equilibrium for baseline PF and MLI rates, the synaptic weight value does not change remarkably (Figure 2, Supplementary Figure 3).

#### Simulation V: PF bundle stimulation at 50 Hz with paired MLI voltage clamp induces LTD

**Figure 3:**
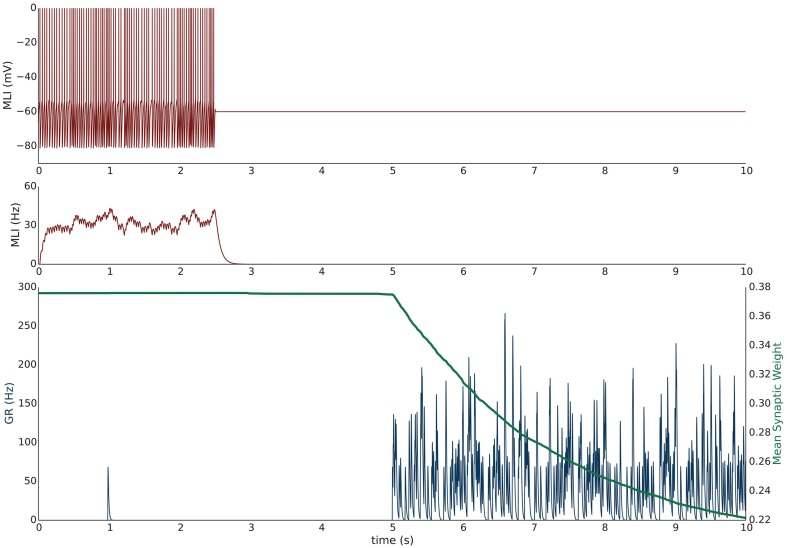
**Simulation V: Voltage clamping MLI induces PF-MLI LTD**. An isolated MLI fires spontaneously for the first 2.5 s of the simulation and is then voltage clamped to −60 mV for the remainder of the simulation. Starting at 5 s, each PF in the bundle fires at 50 Hz. Figure conventions are the same as Figure 1 except that the mean synaptic weight value across all PF synapses converging onto this MLI is shown (green) and only one sample PF trace is shown (blue). Between 2.5 and 5 s, the synaptic weights do not change significantly since the PFs are firing at a low 0.33 Hz. However, once the PFs begin firing at 50 Hz, the synaptic weight decreases rapidly since the normalized value of MLI firing is effectively 0 and below the synaptic weight value. This simulation attempts to reproduce the experimental results of Liu and Cull-Candy (2000).

Simulation V emulates the experimental conditions from Liu and Cull-Candy (2000) used to induce PF-MLI LTD. The simulation models a single isolated MLI with eight PF inputs to emulate the effect of stimulating a bundle of PFs with an electrode *in vitro*. The simulation begins with a 2.5 s baseline period where the PFs and MLI fire at baseline rates of 0.33 Hz and 30 Hz, respectively (Figure 3). At 2.5 s, the MLI is then voltage clamped to −60 mV. At 5 s, the PFs are stimulated to individually fire at approximately 50 Hz (according to Poisson statistics) which continues for 60 s. As before, each 1 s interval starting at 5 s is considered a trial to allow comparison among simulations. Concomitant with the activation of PFs, the synaptic weights decrease since the MLI firing activity is effectively zero, below the value of the synapse weight. Figure 3 depicts these changes, where figure conventions are the same as before except that the weight trace is the mean weight of all PF synapses impinging on the MLI and only one example PF trace is shown. The trajectory of the mean and range of synaptic weights onto this MLI is summarized in **Figure 5**. The synaptic weight asymptotically decreases to the minimum weight value, *w*_0_. The synaptic weight decrease in the simulation is consistent with the observed decrease in synaptic efficacy by Liu and Cull-Candy (2000).

#### Simulation VI: PF bundle burst stimulation with paired MLI current clamp induces LTP

**Figure 4:**
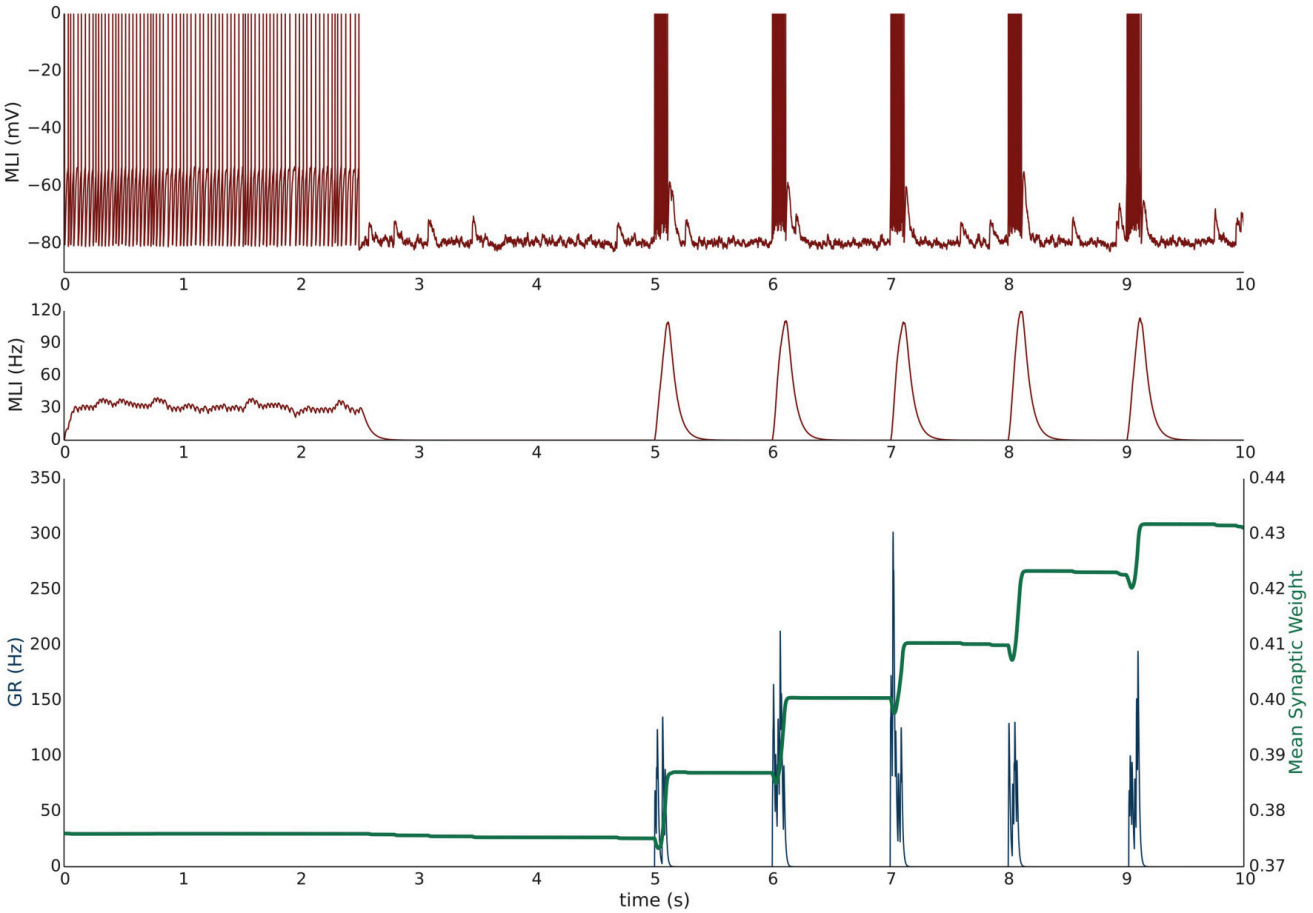
**Simulation VI: Current clamping MLI with PF bursting results in LTP**. An isolated MLI fires spontaneously for the first 2.5 s of the simulation and is then injected with a constant current to keep the membrane potential near −80 mV for the remainder of the simulation. Starting at 5 s, each PF in the bundle fires 100 Hz bursts for 100 ms every 1 s. Figure conventions are the same as Figure 3. Synchronous PF burst firing is sufficient to depolarize the MLI and cause it to fire. During periods where the PFs are active, the MLI firing rate trace rises above the value of the synaptic weight and thus results in an increase in the synaptic weight, i.e., LTP. This simulation attempts to reproduce the experimental results of Smith and Otis (2005).

**Figure 5:**
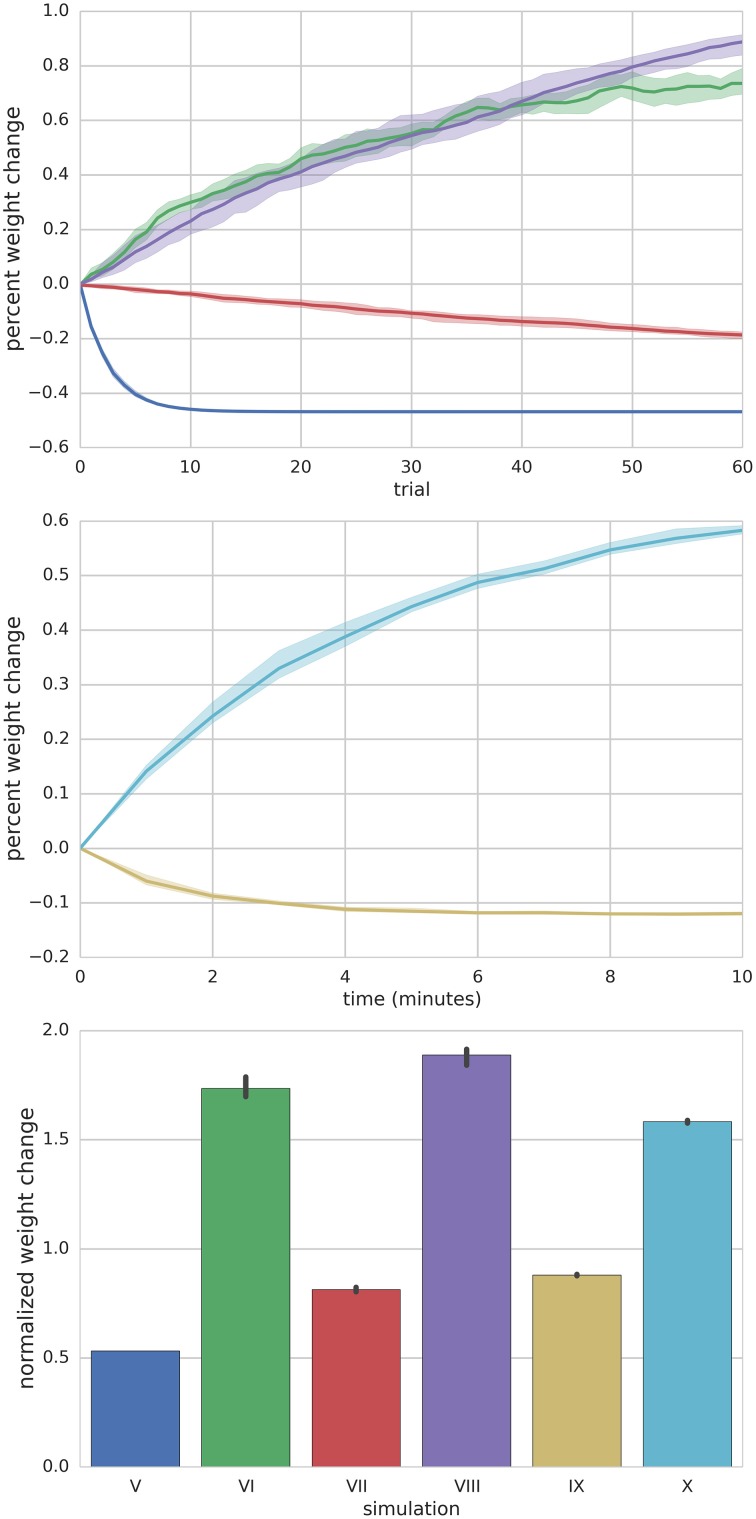
**Summary of Simulations V–X**. Top panel depicts the mean (solid lines) and range (shaded) percent weight change of synaptic weights converging onto the MLI at the end of each trial for simulations V–VIII. Middle panel shows the mean (solid lines) and range (shaded) percent weight change of synaptic weights converging onto the MLI at the end of each minute of simulation time for simulations IX and X. The bottom panel compares the final mean normalized synaptic weights at the end of the simulation for each simulation (bar height) and range of values (black error bars).

Simulation VI models the experimental protocol used by Smith and Otis (2005) to induce PF-driven LTP at PF-MLI synapses. A single isolated, spontaneously firing MLI receiving eight PF inputs is simulated. The simulation begins with the MLI and PFs firing at their baseline rates of 30 and 0.33 Hz, respectively (Figure 4). After 2.5 s, a constant current is injected into the MLI to hold it near −80 mV. Beginning at 5 s, each PF is stimulated to fire at 100 Hz (according to Poisson statistics) for 100 ms every 1 s and continues for 60 s. Despite being injected with a hyperpolarizing current, the cumulative PF input is sufficient to cause the MLI to depolarize and fire action potentials, raising the MLI activity trace from near zero to above the original weight equilibrium values when both PF and MLI fire spontaneously. Thus, the synaptic weights increase during these periods of PF activity and results in a cumulative LTP (Figure 5). Although not apparent in Figure 4, the peak firing rates of MLIs increase along with synaptic weight increases (Supplementary Figure 8).

#### Simulation VII: PF bundle low-frequency stimulation with paired MLI current clamp induces LTD

Simulation VII models a different experimental protocol used by Smith and Otis (2005) which was shown to induce PF-MLI LTD. The simulation protocol is identical to the one described for simulation VI except that each PF is stimulated to produce 2 Hz firing continuously (Supplementary Figure 4) beginning at 5 s. The low frequency spike inputs from all 8 simulated PFs is insufficient to regularly depolarize the MLI. Thus, the MLI activity trace remains zero while the PF activity trace is briefly greater than zero for short periods of time. The cumulative effect is a decrease in the mean PF-MLI synaptic strength, i.e., LTD (Figure 5).

#### Simulation VIII: PF bundle low-frequency stimulation with paired MLI depolarizing current injection induces LTP

Simulation VIII models one of the experimental protocols used by Rancillac and Crépel (2004) which results in PF-MLI LTP. The protocol consists of an induction period of low-frequency (0.33 Hz) stimulation of the PF bundle for 8 min while simultaneously holding the MLI in voltage clamp at −60 mV. After the induction period, the PFs are stimulated to fire at 2 Hz while the MLI is depolarized to 0 mV for 60 s. We hypothesized that during this induction period the synaptic efficacy would decrease from its previous equilibrium value since the PF is somewhat active and the MLI is in voltage clamp. To investigate this in the simulation, we begin the simulation with the MLI in voltage clamp at −60 mV and the synaptic weight at a lower value than previous experiments. The MLI is held in voltage clamp for the first 5 s of the simulation while the PFs fire spontaneously around 0.33 Hz (Supplementary Figure 5). After 5 s, the MLI is injected with current to fire at 50 Hz. Since holding the simulated MLI at 0 mV would not result in spiking and thus a zero value for the activity trace, we chose to inject current to depolarize the neuron to fire at 50 Hz and produce a non-zero activity trace as a surrogate. The result of the simulated protocol is that the MLI activity trace value is greater than the synaptic weight and thus a strengthening of the synapse occurs (Figure 5). This is consistent with the result of Rancillac and Crépel (2004).

#### Simulations IX and X: simulating changes in “basal tone” induces bidirectional changes in synaptic efficacy

Metabotropic 1 glutamate receptors (mGluR_1_) and gamma-aminobutyric acid B receptors (GABA_B_R) found in MLIs are tonically active and their level of activity sets the “basal tone” for PF-MLI EPSC amplitude *in vitro* (Kelly et al., 2009). Simulations IX and X reproduce the effects of modulating the activity of these metabotropic receptors *in vitro* using chemical agonists and antagonists by modifying γ in the synaptic weight update rule. By increasing γ, the MLI activity level needed to surpass the γ*w* threshold for LTP is increased. Thus, if *w* begins near its equilibrium value for baseline PF and MLI activity and γ is increased, *w* will decrease to reach a new equilibrium. In Simulation IX, 8 PFs connected to a MLI all fire spontaneously at baseline rates of 0.33 Hz and 30 Hz, respectively, and the synaptic weights begin near their equilibrium values (Supplementary Figure 6). Starting a 5 s, and continuing for 10 min, γ is raised from γ = 1.0 to γ = 1.5. The result is a gradual decay in the synaptic weight (Figure 5) which is consistent with observed LTD when applying mGluR_1_ and GABA_B_R agonists *in vitro* (Kelly et al., 2009). Conversely, in Simulation X, by decreasing γ from γ = 1.0 to γ = 0.5*w* increases to reach a new equilibrium, reproducing the effects of applying chemical antagonists of applying mGluR_1_ and GABA_B_R antagonists *in vitro* (Kelly et al., 2009) (Supplementary Figure 7).

## Discussion

In this study, we present a mathematical model of plasticity at PF-MLI synapses that describes bidirectional changes in synaptic efficacy as observed *in vitro*. The current model depends only on the pre- and post-synaptic neuronal activity and a dynamic threshold partly determined by a measure of synaptic efficacy. The dynamic threshold enables bidirectional changes in efficacy and is inherently self-stabilizing. We show via computer simulations that the model reproduces similar changes in synaptic efficacy as observed in a variety experimental results. In addition, we simulate novel protocols which serve as predictions to be validated by future experimental investigation.

### Biological mechanisms of the model

The model presented in this study is a *phenomenological* description of plasticity that is a function of PF and MLI spiking activity and a measure of synaptic efficacy. This model serves as a high-level surrogate for describing plasticity until the detailed biological mechanisms are modeled directly. In this section, we speculate on what the underlying mechanisms are which give rise to this phenomenological description.

Plasticity at the PF-MLI synapses involves several mechanisms that produce both pre- and post-synaptic changes (Liu and Cull-Candy, 2000; Rancillac and Crépel, 2004; Bender et al., 2009). While we mainly focus on evidence for post-synaptic plasticity, it is expected that this model also accounts for pre-synaptic changes in synaptic efficacy in physiologically realistic conditions. Post-synaptic PF-MLI plasticity involves changes in the AMPA receptor (AMPAR) phenotype composition (Liu and Cull-Candy, 2000). Stellate-type MLIs, and presumably basket-type MLIs, express glutamate receptor 2 (GluR2) lacking calcium-permeable AMPARs (CP-AMPARS) and GluR2-containing calcium-impermeable AMPARs (CI-AMPAR) and have been shown to make an activity dependent switch from CP- to CI-AMPARs (Liu and Cull-Candy, 2000, 2002; Kelly et al., 2009). CP-AMPARs admit more charge at negative potentials (Liu and Cull-Candy, 2000) so a switch from CP- to CI-AMPARs results in a functional decrease in the strength of this synapse. Thus, the mixture of CP/CI-AMPARs at the post-synaptic density determines the excitatory post-synaptic potential (EPSC) amplitude, or “basal tone,” of the synapse. Additionally, the level of tonic activation of mGluR_1_ and GABA_B_ receptors influence the basal tone of the synapse (Kelly et al., 2009). Both activity dependent LTP and LTD at this synapse are observed and are post-synaptic calcium signaling dependent (Liu and Cull-Candy, 2000; Rancillac and Crépel, 2004; Smith and Otis, 2005; Sun and June Liu, 2007).

We hypothesize that activity-dependent post-synaptic Ca^2+^ transients initiate changes in synaptic efficacy. These transients could induce both LTD and LTP dependent on a dynamic cytosolic Ca^2+^ threshold which could be reflected in the level of intracellular calcium stores. In the model proposed here, the dynamic threshold is captured by γ*w*, where *w* captures the strength of the synapse in terms of CP- and CI-AMPAR makeup, and γ captures the effects of basal activity levels of metabotropic receptors involved in AMPAR phenotype composition. CP-AMPARs are one source of calcium influx. Indeed, the change in AMPAR phenotype composition could provide one mechanism for governing bidirectional changes in plasticity. The upper limit on synaptic efficacy could be governed by the dependence on sufficient amounts of calcium influx simply to maintain the phenotype composition of the synapse since stronger synapses may require increasingly greater post-synaptic calcium concentrations simply to maintain or increase the strength of the synapse during periods of PF activation. This limit may be further enhanced by partial block of calcium influx through CP-AMPARs during physiologic activation due to intracellular polyamines (Bats et al., 2013). The lower limit on synaptic efficacy could be governed by the dependence of the CP- to CI-AMPAR switch on calcium influx through CP-AMPARs (Liu and Cull-Candy, 2000, 2005; Gardner et al., 2005).

NMDARs are another source of calcium influx for signaling changes in plasticity which are MLI activity dependent. Indeed, blocking NMDARs prevents LTP and even uncovers some LTD during PF stimulation *in vitro* (Rancillac and Crépel, 2004; Smith and Otis, 2005). MLI NMDARs are located extrasynaptically (Clark and Cull-Candy, 2002) and can be activated by a single PF firing at a sufficiently high frequency (Nahir and Jahr, 2013). Since PFs fire high-frequency bursts in physiologically realistic conditions (Chadderton et al., 2004; van Beugen et al., 2013), PFs likely activity NMDARs *in vivo* when firing bursts. Furthermore, climbing fibers (CFs) activate MLIs exclusively via glutamate spillover (Szapiro and Barbour, 2007) and CF mediated EPSCs have a significant NMDAR-mediated component (Coddington et al., 2013). Thus, CFs may play a special role in gating calcium influx and biasing plasticity toward LTP *in vivo*. This is consistent with adaptive filter models of cerebellar learning which require correlated PF and CF firing to induce LTP at these synapses (Dean et al., 2010).

The proposed model is also a function of PF and MLI activity which is a normalized, unit-less trace of the spiking activity of these neurons. Calcium signals could be the common mechanism of conveying this activity. MLI somatic calcium concentrations change slowly as a function of the firing rate of these neurons (Franconville et al., 2011) and dendritic calcium concentrations are regulated by somatic spikes (Myoga et al., 2009). Thus, the firing rate of MLIs could produce a global time-varying calcium concentration in the dendrites of MLIs. Similarly, PF traces could be implemented using calcium signals since physiological PF firing results in prolonged glutamate conductances, and thus calcium influx, at MLI synapses (Carter and Regehr, 2000). Furthermore, changes in PF-MLI synapses are input-specific due to localized synaptic Ca^2+^ signaling in MLI dendrites (Soler-Llavina and Sabatini, 2006). Thus, a synapse specific trace of PF activity may use a sustained level of glutamate at the synapse and local calcium concentrations in the post-synaptic membrane.

### Limitations of the model

The model is limited to describing plasticity at the level of pre- and post-synaptic activities and is thus unable to simulate certain physiological conditions from past experimental protocols. In particular, calcium currents from AMPARs and NMDARs are not directly accounted for in this model and their influence is based on their collective ability to depolarize the MLI. This makes simulating chemical NMDA channel block and voltage-dependent calcium conductances, such as holding the MLI in voltage clamp at 0 mV, (as in Simulation VIII) impossible. In addition, the effect of modulating metabotropic receptor activities and their downstream effects cannot be modeled directly. The model is also unrealistic in that changes in synaptic efficacy happen instantaneously whereas changes *in vitro* and *in vivo* continue to take place for several minutes after stimulation (Rancillac and Crépel, 2004; Smith and Otis, 2005).

### Related models of plasticity

BCM theory (Bienenstock et al., 1982) is a model of plasticity used to describe activity dependent synaptic changes in the visual cortex which also employs a dynamic threshold to induce bidirectional changes in synaptic efficacy. Using the BCM model in lieu of the gated steepest descent model would not reproduce all of the experimental results described here. This can be seen using Simulation V as an example. Using a trace of MLI spiking for the post-synaptic activity in the BCM weight update equation would result in no weight change when the MLI is held in current clamp since this trace is effectively zero and all other terms of the weight update equation are multiplied by this term.

A number of other models of cerebellar learning have either explicitly or implicitly modeled learning at PF-MLI synapses, but most of these models were proposed before any experimental evidence describing plasticity at this synapse existed. Kenyon (1997) proposed a model where changes in PF-PKJ synapses are consolidated into long-term memories at PF-MLI synapses, but the weight update equation used doesn't appear to be consistent with experimental evidence. Albus (1971) also predicted learned changes to PF-MLI synapses but suggested that CF inputs act to weaken PF-MLI synapses, similar to PF-PKJ synapses. Adaptive filter models of cerebellar learning have been proposed that use positive and negative values for adaptive weights which implicitly defines plasticity at MLI synapses that is complementary and synergistic to PF-PKJ learning (Dean et al., 2010).

Finally, a modified gated steepest descent learning rule has also been used to model plasticity at the synapses formed by mossy fibers onto neurons in the deep cerebellar nuclei/vestibular (Yamazaki et al., 2015, Supplementary Information, Equation 9). Simulations using this model reproduce post-training memory consolidation in learned gain changes of the optokinetic response.

### Extending the model

Concomitant PF and climbing fiber (CF) activation leads to a drastic increase in the PF-MLI receptive field (RF) and subsequent PF stimulation alone leads to a decrease in the PF-MLI RF (Jörntell and Ekerot, 2002, 2003, 2011). The increased RF could be due to activation of electrically silent synapses, however the mechanisms governing this process are not understood. Assuming this *in vitro* model holds under *in vivo* conditions, one way to augment the current model is to include a separate equation for plasticity at electrically silent synapses that requires concomitant activation of PF and CF input in order for a synapse to become electrically active. In simulations, this would result in an increase in the number of synapses that depolarize the target MLI and an effective increase in the RF size.

CFs may also influence plasticity at active synapses in two ways: indirectly, by increasing MLI firing rates thus favoring LTP, and directly, by modulating the threshold for plasticity through γ—capturing the effects of glutamate spillover and changes in post-synaptic calcium concentration due to activation of NMDARs and CP-AMPARs. Note that this would be a separate mechanism than modulating metabotropic receptors to modulate γ. This could be implemented where γ is a function of the activity of CFs or the spillover of glutamate from CF inputs to the MLI. When CFs are inactive (active) or the volume of glutamate spillover is low (high), γ would be high (low). This mechanism would bias PF-MLI synapses with concomitant PF and CF activation toward potentiation by lowering the threshold in favor of LTP. Subsequently, if the PF were active without concomitant CF activation, the synapse might weaken since γ and thus γ*w* is higher than before. This could be one mechanism for observing decreases in PF receptive field sizes with PF stimulation subsequent to a PF+CF protocol which results in PF receptive field increases (Jörntell and Ekerot, 2002). Homeostatic plasticity (Turrigiano, 2012) such as synaptic scaling may be another mechanism that reduces receptive field sizes over time or causes electrically active synapses to become inactive. Taken together, we predict that the extended model would reproduce the RF changes observed in the *in vivo* experiments performed by Jorntell and Ekerot.

The model can also be extended to model calcium concentrations directly. Similar models based on BCM theory (Bienenstock et al., 1982) have been extended to model plasticity as a function of calcium concentrations which include influences from both AMPARs, NMDARs and action potentials (Shouval et al., 2002; Yeung et al., 2004). Indeed, the form of the equations governing plasticity as a function of calcium concentrations in these models is similar to the model presented here. The model presented in Shouval et al. (2002) could be modified in the following ways to capture synaptic plasticity at PF-MLI synapses. The calcium influx would be a function of the proportion of CP-AMPARs which changes with the synaptic strength as well as a function of spillover glutamate from high frequency PF bursts and CF volumergic transmission separately. In addition, the glutamate binding dynamics from PFs would be modeled which results in prolonged calcium influx. “Assumption 3” of Shouval et al. (2002) is similar to the slowly changing calcium concentrations in MLIs due to spiking activity (Myoga et al., 2009; Franconville et al., 2011). The model would have to be further augmented to capture the effect of metabotropic receptors on plasticity. A simulation of the described model should reflect predictions made by our model, but with more physiological detail and will allow for more direct simulation of NMDA channel block and voltage-dependent calcium currents under voltage-clamp conditions.

### Implications of the model

Historically, network models of the cerebellum have focused on PF-PKJ synapses as the locus of learning and memory in the cerebellar cortex. However, effective cerebellar learning is likely the result of distributed plasticity across synapse types in the cerebellum (Gao et al., 2012; Mapelli et al., 2015). MLI synapses have been previously suggested as one such location (Jörntell et al., 2010; D'Angelo, 2014).

The learning rule for PF-MLI synapses presented here takes the form of Grossberg's *outstar* learning rule (Grossberg, 1968). In accordance with this learning rule, granule cells (GRs) will “sample” the responses of their MLI targets and adjust their synaptic weights to “track” the normalized MLI activity values when PF synapses are active. If a distributed pattern of activity across the MLI population is repeatedly present concomitant with a GR encoded pattern of some stimulus, the PFs will adapt their weights so that the GR activity pattern alone will reproduce the MLI activity pattern. Thus, it may be the role of the CF inputs to the MLIs to act as a supervised learning signal and to initialize the distributed pattern of activity across the MLIs and to bias synaptic changes toward strengthening. Subsequently, the PF-MLI response will be learned and reproduced associatively.

The role of MLI-MLI plasticity may be to normalize and contrast enhance the MLI population response and to induce competition among MLIs in the distributed pattern of activity across the local MLI population. Furthermore, MLI-PKJ plasticity would enable learning combinations of MLI activity patterns to produce appropriate PKJ responses. Finally, PF-PKJ plasticity may act synergistically with changes in feedforward inhibition from MLIs, although the two inputs could have different time courses for affecting PKJ responses. Extending the model to account for the interplay between CFs and PF-MLI plasticity may be necessary before network simulations of the cerebellum using this model of plasticity would be illuminating.

### Interpretation of experimental results

A number of *in vitro* experimental protocols used to induce plasticity at PF-MLI synapses can be described in terms of the model presented in this study which also correctly predicts the experimental results. We present this interpretation and speculate on some of the biological mechanisms responsible for plasticity in each case. Table 4 summarizes selected experiments and their interpretations in terms of this model.

**Table 4:** **Summary of experimental results**.

**Simulation**	**References**	**Experimental protocol**	**Result**	**Interpretation**
V	Liu and Cull-Candy, 2000	*In vitro*, MLI voltage clamped at −60 mV. 300 PF stimuli delivered @ 50 Hz. Bicuculline, picrotoxin, D-APV5.	LTD	Holding the MLI in voltage clamp prevents its spontaneous activity and prevents inward Ca^2+^ currents (↓**MLI**). PF stimulation at 50 Hz increases PF activity above baseline (↑**PF**). **Δw =** ↑**PF(**↓**MLI-w)** < **0**
VI	Smith and Otis, 2005	*In vitro*, MLI current clamped at −80 mV. PFs stimulated (3–10 PFs) with 10 pulses at 100 Hz every 3 s for 5 min. Picrotoxin in bath.	LTP	Sufficient PF stimulation (↑**PF**) increases MLI activity (↑**MLI**) since current clamp allows the membrane potential to fluctuate. **Δw** = ↑**PF(**↑**MLI-w)** > **0**
VII	Smith and Otis, 2005	*In vitro*, MLI current clamped at −80 mV. PFs stimulated (3–10 PFs) with at 1 Hz for 5 min. Picrotoxin in bath.	LTD	Low frequency PF stimulation is a slight increase in PF activity (↑**PF**) but insufficient to increase MLI activity which is held in current clamp (↓**MLI**) **Δw** = ↑**PF(**↓**MLI-w)** < **0**
Similar to IV	Rancillac and Crépel, 2004	*In vitro*, MLI voltage clamp at −60 mV. Bicuculline in bath. Induction protocol of PF stimulation at 0.33 Hz for 8 min, then PF stimulated at 2 Hz for 60 s.	LTP/-/LTD	The induction protocol initially shifts ↓w to a new value w^*^ since ↓MLI. The plasticity protocol then increases PF activity (↑**PF**). **Δw** = ↑PF(MLI-w^*^) ≈ **0**
VIII	Rancillac and Crépel, 2004	*In vitro*, MLI voltage clamp at −60 mV. Bicuculline in bath. Induction protocol of PF stimulation at 0.33 Hz for 8 min, then PF stimulated at 2 Hz for 60 s with a paired MLI depolarization at 0 mV	LTP/-	The induction protocol initially shifts ↓w to a new value w^*^ since ↓MLI. The plasticity protocol then increases PF activity (↑**PF**) and admits more Ca^2+^ possibly via NMDARs and VGCCs (↑**MLI**). **Δw** = ↑**PF(**↑MLI-w^*^) > **0**
—	Sun and June Liu, 2007	*In vitro*, MLI held at −70 mV during stimulation followed by brief depolarization to 0 mV. GYKI in path (AMPAR blocker). PF 4 stimuli @ 50 Hz, 100 sweeps.	LTD	High frequency PF bursts (↑**PF**) paired with brief MLI depolarization, leads to spillover activation of NMDARs and limited Ca^2+^ influx via NMDARs. Since CP-AMPARs are blocked and MLI spontaneous activity is prevented via voltage clamp, the overall Ca^2+^ transient is below threshold (↓**MLI**). **Δw** = ↑**PF(**↓**MLI-w)** < **0**
IX	Kelly et al., 2009	*In vitro*, MLI voltage clamped at −60 mV. D-APV5, bicuculline in bath. Either DHPG (mGluR1 agonist) or baclofen (GABA_B_R agonist) added.	LTD	Increasing mGluR Group I activity (↑mGluR) can be interpreted as increasing PF activity (↑**PF**). Additionally, ↑mGluR may directly increase the intracellular Ca^2+^ threshold required for plasticity (↑**w**). Increasing GABA_B_R (↑GABA_B_R) enhances mGluR activity, and a similar result holds. **Δw** = ↑**PF(MLI-**↑**w)** < **0**
X	Kelly et al., 2009	*In vitro*, MLI voltage clamped at −60 mV. D-APV5, bicuculline in bath. Either LY367385 (mGluR1 antagonist) or CGP62349 (GABA_B_R antagonist) added.	LTP	Decreasing mGluR activity adjusts the “basal tone” by shifting the intracellular Ca^2+^ threshold for plasticity (↓**w**). Ambient glutamate allows some Ca^2+^ to still flow into the MLI. Since GABA_B_R enhances mGluR activity, ↓GABA_B_R causes ↓mGluR, thus ↓**w**. **Δw** = **PF(MLI-**↓**w)** > **0**

Early on, it was shown that high frequency stimulation of PFs while holding the MLI in voltage clamp at −60 mV induces a switch from CP- to CI-AMPARs (Liu and Cull-Candy, 2000), i.e., LTD. This result is predicted by the model since holding the MLI in voltage clamp decreases its activity relative to baseline ($↓\bar{\text{MLI}}\left(t\right)$), and stimulating PFs increases their activity relative to baseline ($↑\bar{\text{PF}}\left(t\right)$); i.e., $\Delta w\propto ↑\bar{\text{PF}}\left(t\right)\left[↓\bar{\text{MLI}}\left(t\right)-\gamma w\right]<0$ where $\bar{\text{MLI}}\left(t\right)<\gamma w$. Chelating post-synaptic Ca^2+^ prevented the switch in AMPAR phenotype and resulted in no change in synaptic efficacy, supporting the idea that the mechanism of plasticity is calcium signaling dependent. Similarly, a separate study showed that a 30 Hz PF stimulation with the MLI held in voltage clamp leads to pre-synaptic LTD that is also dependent on post-synaptic Ca^2+^ influx (Soler-Llavina and Sabatini, 2006), suggesting a complementary form of LTD. While this is a different mechanism, it is consistent with the model prediction by the same reasoning.

In somewhat more realistic physiological conditions, high frequency PF burst stimulation was shown to induce LTP *in vitro* (Smith and Otis, 2005). This was demonstrated in two ways. In the first method, MLIs were held in current clamp at −80 mV while PFs were stimulated to fire brief high frequency bursts at 100 Hz. The bath contained picrotoxin to block inhibitory currents into the MLI. Following the plasticity protocol, LTP was measured directly by observing an increase in MLI spike firing in response to PF input compared to control conditions. In the second method, synaptic changes were induced indirectly by stimulating the PFs according to the same protocol but in a bath without picrotoxin and then recording responses from PKJs. After the protocol, PKJs initially had a higher firing rate due to PF-PKJ LTP, followed by a period of spike depression caused by inhibition, presumably from increased MLI feedforward inhibition. Additional experimental evidence suggests increased depression appears to be due to PF-MLI potentiation and not from MLI-PKJ potentiation. The model predicts LTP in these experiments by $\Delta w\propto ↑\bar{\text{PF}}\left(t\right)\left[↑\bar{\text{MLI}}\left(t\right)-\gamma w\right]>0$ with $\bar{\text{MLI}}\left(t\right)>\gamma w$ since the membrane potential of the MLI is able to fluctuate during PF stimulation in contrast to the protocol used in Liu and Cull-Candy (2000) where it is voltage clamped. Using the same LTP-inducing protocol in the presence of NMDAR antagonists, LTP is abolished and some LTD is uncovered (Smith and Otis, 2005). NMDARs are located extrasynaptically and can be activated by a high frequency train of PF stimulation (Carter and Regehr, 2000; Clark and Cull-Candy, 2002). This suggests the 100 Hz stimulation caused spillover activation of NMDARs and that this is important for LTP, presumably due to Ca^2+^ influx since chelating post-synaptic Ca^2+^ also blocked LTP (Smith and Otis, 2005). Using a low frequency stimulation protocol consisting of PF stimulation at 1 Hz for 5 min, PF-MLI LTD is observed both directly and indirectly (Smith and Otis, 2005). In the indirect case when the MLI is held in current clamp, the stimulus may be insufficient to perturb the MLI membrane potential significantly or to activate extrasynaptic NMDARs, thus the current clamp acts similar to voltage clamp as in previous experiments. The model also predicts LTD, i.e., $\Delta w\propto ↑\bar{\text{PF}}\left(t\right)\left[↓\bar{\text{MLI}}\left(t\right)-\gamma w\right]<0$.

Sun and June Liu (2007) investigated the role of NMDARs in the CP- to CI-AMPAR switch. To induce this change, MLIs were held in voltage clamp at −60 mV while chemically blocking AMPARs; PFs were stimulated to produce high frequency bursts that activated NMDARs and were paired with fast, 1 ms, MLI depolarizations to 0 mV to release NMDAR Mg^2+^ block. A Ca^2+^ dependent CP- to CI-AMPAR switch was observed, suggesting that Ca^2+^ entry through NMDARs provide an additional pathway to induce plasticity at the synapse. While Ca^2+^ enters through NMDARs during the brief depolarization, it is insufficient to signal LTP since the normal spiking activity of the MLI, and thus cytosolic calcium concentration, is reduced by voltage clamp. The model would reflect this as $\Delta w\propto ↑\bar{\text{PF}}\left(t\right)\left[↓\bar{\text{MLI}}\left(t\right)-\gamma w\right]<0$ where $\bar{\text{MLI}}\left(t\right)<\gamma w$.

Rancillac and Crépel (2004) found that holding the MLI in voltage clamp at −60 mV and stimulating PFs at 2 Hz resulted in a mix of LTP and LTD at the PF-MLI synapse. One explanation for the mix of LTP/LTD may be the result of the induction protocol used which held the MLI in voltage clamp at −60 mV while stimulating the PFs at 0.33 Hz for several minutes; this could decrease the synaptic strength and/or the dynamic threshold down during this period. During the experiment, the MLI activity is compared to the threshold for synaptic plasticity—for some synapses, the low activation could be sufficient to surpass the threshold ($\bar{\text{MLI}}\left(t\right)>\gamma w$) and not for others ($\bar{\text{MLI}}(t)<\gamma w)$. Thus, on average $\Delta w\propto ↑\bar{\text{PF}}\left(t\right)\left[\bar{\text{MLI}}\left(t\right)-\gamma w\right]\approx 0$. In contrast, when repeating this stimulation and pairing it with MLI depolarization at 0 mV more cells underwent LTP, indicating that post-synaptic activity plays a role in plasticity, i.e., $\Delta w\propto ↑\bar{\text{PF}}\left(t\right)\left[↑\bar{\text{MLI}}\left(t\right)-\gamma w\right]>0$. This form of LTP was independent of cAMP but required NO production. In another experiment stimulating PFs at 8 Hz while holding the MLI in voltage clamp induced a mix of LTP or no change in tested synapses, but part of the LTP was cAMP dependent (Rancillac and Crépel, 2004). This last result in consistent with (Bender et al., 2009) which showed pre-synaptic LTP dependent on cAMP through a similar induction protocol. These results reveal the complexity of synaptic plasticity at the PF-MLI synapse consisting of both pre- and post-synaptic mechanisms induced under artificial physiological conditions.

Kelly et al. (2009) found that activation of both mGluRs and CP-AMPARs is necessary and sufficient to drive the CP- to CI-AMPAR subunit switch and that activation of GABA_B_R enhances mGluR activity. Adding mGluR_1_ agonists to the *in vitro* preparation results in LTD at the synapse. A similar effect is seen when adding GABA_B_R agonists to the bath. Assuming metabotropic receptors act to directly modulate the post-synaptic cytosolic calcium threshold used for bidirectional changes in plasticity, the effects of up-regulating these metabotropic receptor activities can be seen as increasing γ in the model, i.e., $\Delta w\propto \bar{\text{PF}}\left(t\right)\left[\bar{\text{MLI}}\left(t\right)-↑\gamma w\right]<0$. Similarly, adding mGluR_1_ and GABA_B_R antagonists results in LTP which can be interpreted as ↓γ.

## Conclusion

In summary, we have shown that a simple mathematical model of plasticity at PF-MLI synapses captures most reported phenomena of plasticity at this synapse. We carried out several numerical simulations of this plasticity rule implemented at PF-MLI synapses using a previously published leaky integrate-and-fire model of MLIs. We showed that this model reproduces the experimental results of several plasticity inducing protocols reported in the literature. Additionally, we simulated several novel protocols that have not been published to serve as predictions for the model. Finally, we speculated on the biophysical mechanisms governing plasticity at this synapse and the implications of this form of plasticity on the network function of the cerebellar cortex.

## Funding

The authors thank the Japan Society for the Promotion of Science (#SP13031) and the U.S. National Science Foundation (#OISE-1308822) for making this collaboration possible. WL and RH-N gratefully acknowledge funding from the U.S. Office of Naval Research (#N00014-12-1-0588).

### Conflict of interest statement

The authors declare that the research was conducted in the absence of any commercial or financial relationships that could be construed as a potential conflict of interest.

## References

[B1] Albus J. S. (1971). A theory of cerebellar function. Math. Biosci. 10, 25–61. 10.1016/0025-5564(71)90051-4

[B2] Bats C. Farrant M. Cull-Candy S. G. (2013). A role of TARPs in the expression and plasticity of calcium-permeable AMPARs: evidence from cerebellar neurons and glia. Neuropharmacology 74, 76–85. 10.1016/j.neuropharm.2013.03.037 23583927 PMC3751754

[B3] Bender V. A. Pugh J. R. Jahr C. E. (2009). Presynaptically expressed long-term potentiation increases multivesicular release at parallel fiber synapses. J. Neurosci. 29, 10974–10978. 10.1523/JNEUROSCI.2123-09.2009 19726655 PMC2775459

[B4] Bienenstock E. L. Cooper L. N. Munro P. W. (1982). Theory for the development of neuron selectivity: orientation specificity and binocular interaction in visual cortex. J. Neurosci. 2, 32–48. 7054394 10.1523/JNEUROSCI.02-01-00032.1982 PMC6564292

[B5] Brunel N. Hakim V. Isope P. Nadal J. P. Barbour B. (2004). Optimal information storage and the distribution of synaptic weights: perceptron versus Purkinje cell. Neuron 43, 745–757. 10.1016/j.neuron.2004.08.023 15339654

[B6] Carter A. G. Regehr W. G. (2000). Prolonged synaptic currents and glutamate spillover at the parallel fiber to stellate cell synapse. J. Neurosci. 20, 4423–4434. 10844011 10.1523/JNEUROSCI.20-12-04423.2000 PMC6772456

[B7] Carter A. G. Regehr W. G. (2002). Quantal events shape cerebellar interneuron firing. Nat. Neurosci. 5, 1309–1318. 10.1038/nn970 12411959

[B8] Cerminara N. L. Rawson J. A. (2004). Evidence that climbing fibers control an intrinsic spike generator in cerebellar Purkinje cells. J. Neurosci. 24, 4510–4517. 10.1523/JNEUROSCI.4530-03.2004 15140921 PMC6729399

[B9] Chadderton P. Margrie T. W. Häusser M. (2004). Integration of quanta in cerebellar granule cells during sensory processing. Nature 428, 856–860. 10.1038/nature02442 15103377

[B10] Chen Z. (2007). Correlative Learning: A Basis for Brain and Adaptive Systems. Hoboken, NJ: Wiley-Interscience.

[B11] Clark B. A. Cull-Candy S. G. (2002). Activity-dependent recruitment of extrasynaptic NMDA receptor activation at an AMPA receptor-only synapse. J. Neurosci. 22, 4428–4436. 12040050 10.1523/JNEUROSCI.22-11-04428.2002 PMC6758796

[B12] Coddington L. T. Rudolph S. Vande Lune P. Overstreet-Wadiche L. Wadiche J. I. (2013). Spillover-mediated feedforward inhibition functionally segregates interneuron activity. Neuron 78, 1050–1062. 10.1016/j.neuron.2013.04.019 23707614 PMC3733564

[B13] D'Angelo E. (2014). The organization of plasticity in the cerebellar cortex: from synapses to control. Prog. Brain Res. 210, 31–58. 10.1016/B978-0-444-63356-9.00002-9 24916288

[B14] Dean P. Porrill J. Ekerot C. F. Jörntell H. (2010). The cerebellar microcircuit as an adaptive filter: experimental and computational evidence. Nat. Rev. Neurosci. 11, 30–43. 10.1038/nrn2756 19997115

[B15] Eccles J. C. Ito M. Szentaìgothai J. N. (1967). The Cerebellum as a Neuronal Machine. Berlin, New York, NY: Springer-Verlag.

[B16] Franconville R. Revet G. Astorga G. Schwaller B. Llano I. (2011). Somatic calcium level reports integrated spiking activity of cerebellar interneurons *in vitro* and *in vivo*. J. Neurophysiol. 106, 1793–1805. 10.1152/jn.00133.2011 21734102

[B17] Gabbiani F. Midtgaard J. Knöpfel T. (1994). Synaptic integration in a model of cerebellar granule cells. J. Neurophysiol. 72, 999–1009. 7527078 10.1152/jn.1994.72.2.999

[B18] Gao Z. van Beugen B. J. De Zeeuw C. I. (2012). Distributed synergistic plasticity and cerebellar learning. Nat. Rev. Neurosci. 13, 619–635. 10.1038/nrn3312 22895474

[B19] Gardner S. M. Takamiya K. Xia J. Suh J. G. Johnson R. Yu S.. (2005). Calcium-permeable AMPA receptor plasticity is mediated by subunit-specific interactions with PICK1 and NSF. Neuron 45, 903–915. 10.1016/j.neuron.2005.02.026 15797551

[B20] Gerstner W. Kistler W. M. (2002). Spiking Neuron Models: Single Neurons, Populations, Plasticity. Cambridge, UK; New York, NY: Cambridge University Press.

[B21] Goodman D. F. Brette R. (2009). The brian simulator. Front. Neurosci. 3, 192–197. 10.3389/neuro.01.026.2009 20011141 PMC2751620

[B22] Grossberg S. (1968). Some nonlinear networks capable of learning a spatial pattern of arbitrary complexity. Proc. Natl. Acad. Sci. U.S.A. 59, 368–372. 10.1073/pnas.59.2.368 16591608 PMC224680

[B23] Grossberg S. (1969). On learning of spatiotemporal patterns by networks with ordered sensory and motor components. 1. Excitatory components of the cerebellum. Stud. Appl. Math. 48, 105–132. 10.1002/sapm1969482105

[B24] Häusser M. Clark B. A. (1997). Tonic synaptic inhibition modulates neuronal output pattern and spatiotemporal synaptic integration. Neuron 19, 665–678. 10.1016/S0896-6273(00)80379-7 9331356

[B25] Heiney S. A. Kim J. Augustine G. J. Medina J. F. (2014). Precise control of movement kinematics by optogenetic inhibition of Purkinje cell activity. J. Neurosci. 34, 2321–2330. 10.1523/JNEUROSCI.4547-13.2014 24501371 PMC3913874

[B26] Ito M. (2006). Cerebellar circuitry as a neuronal machine. Prog. Neurobiol. 78, 272–303. 10.1016/j.pneurobio.2006.02.006 16759785

[B27] Ito M. Yamaguchi K. Nagao S. Yamazaki T. (2014). Long-term depression as a model of cerebellar plasticity. Prog. Brain Res. 210, 1–30. 10.1016/B978-0-444-63356-9.00001-7 24916287

[B28] Jirenhed D. A. Bengtsson F. Hesslow G. (2007). Acquisition, extinction, and reacquisition of a cerebellar cortical memory trace. J. Neurosci. 27, 2493–2502. 10.1523/JNEUROSCI.4202-06.2007 17344387 PMC6672498

[B29] Jörntell H. Bengtsson F. Schonewille M. De Zeeuw C. I. (2010). Cerebellar molecular layer interneurons - computational properties and roles in learning. Trends Neurosci. 33, 524–532. 10.1016/j.tins.2010.08.004 20869126

[B30] Jörntell H. Ekerot C. F. (2002). Reciprocal bidirectional plasticity of parallel fiber receptive fields in cerebellar Purkinje cells and their afferent interneurons. Neuron 34, 797–806. 10.1016/S0896-6273(02)00713-4 12062025

[B31] Jörntell H. Ekerot C. F. (2003). Receptive field plasticity profoundly alters the cutaneous parallel fiber synaptic input to cerebellar interneurons *in vivo*. J. Neurosci. 23, 9620–9631. 14573542 10.1523/JNEUROSCI.23-29-09620.2003 PMC6740478

[B32] Jörntell H. Ekerot C. F. (2011). Receptive field remodeling induced by skin stimulation in cerebellar neurons *in vivo*. Front. Neural Circuits 5:3. 10.3389/fncir.2011.00003 21427779 PMC3049319

[B33] Kelly L. Farrant M. Cull-Candy S. G. (2009). Synaptic mGluR activation drives plasticity of calcium-permeable AMPA receptors. Nat. Neurosci. 12, 593–601. 10.1038/nn.2309 19377472

[B34] Kenyon G. T. (1997). A model of long-term memory storage in the cerebellar cortex: a possible role for plasticity at parallel fiber synapses onto stellate/basket interneurons. Proc. Natl. Acad. Sci. U.S.A. 94, 14200–14205. 10.1073/pnas.94.25.14200 9391177 PMC28457

[B35] Lachamp P. M. Liu Y. Liu S. J. (2009). Glutamatergic modulation of cerebellar interneuron activity is mediated by an enhancement of GABA release and requires protein kinase A/RIM1alpha signaling. J. Neurosci. 29, 381–392. 10.1523/JNEUROSCI.2354-08.2009 19144838 PMC2775555

[B36] Lennon W. Hecht-Nielsen R. Yamazaki T. (2014). A spiking network model of cerebellar Purkinje cells and molecular layer interneurons exhibiting irregular firing. Front. Comput. Neurosci. 8:157. 10.3389/fncom.2014.00157 25520646 PMC4249458

[B37] Liu S. J. Cull-Candy S. G. (2002). Activity-dependent change in AMPA receptor properties in cerebellar stellate cells. J. Neurosci. 22, 3881–3889. 12019307 10.1523/JNEUROSCI.22-10-03881.2002 PMC6757644

[B38] Liu S. J. Cull-Candy S. G. (2005). Subunit interaction with PICK and GRIP controls Ca^2+^ permeability of AMPARs at cerebellar synapses. Nat. Neurosci. 8, 768–775. 10.1038/nn1468 15895086

[B39] Liu S. J. Savtchouk I. (2012). Ca^(2+)^ permeable AMPA receptors switch allegiances: mechanisms and consequences. J. Physiol. 590, 13–20. 10.1113/jphysiol.2011.213926 21893602 PMC3300041

[B40] Liu S. Q. Cull-Candy S. G. (2000). Synaptic activity at calcium-permeable AMPA receptors induces a switch in receptor subtype. Nature 405, 454–458. 10.1038/35013064 10839540

[B41] Mapelli L. Pagani M. Garrido J. A. D'Angelo E. (2015). Integrated plasticity at inhibitory and excitatory synapses in the cerebellar circuit. Front. Cell. Neurosci. 9:169. 10.3389/fncel.2015.00169 25999817 PMC4419603

[B42] Marr D. (1969). A theory of cerebellar cortex. J. Physiol. 202, 437–470. 10.1113/jphysiol.1969.sp008820 5784296 PMC1351491

[B43] McCormick D. A. Wang Z. Huguenard J. (1993). Neurotransmitter control of neocortical neuronal activity and excitability. Cereb. Cortex 3, 387–398. 10.1093/cercor/3.5.387 7903176

[B44] Midtgaard J. (1992). Membrane properties and synaptic responses of Golgi cells and stellate cells in the turtle cerebellum *in vitro*. J. Physiol. 457, 329–354. 10.1113/jphysiol.1992.sp019381 1338460 PMC1175734

[B45] Miyashita Y. Nagao S. (1984). Contribution of cerebellar intracortical inhibition to Purkinje cell response during vestibulo-ocular reflex of alert rabbits. J. Physiol. 351, 251–262. 10.1113/jphysiol.1984.sp015243 6611408 PMC1193115

[B46] Myoga M. H. Beierlein M. Regehr W. G. (2009). Somatic spikes regulate dendritic signaling in small neurons in the absence of backpropagating action potentials. J. Neurosci. 29, 7803–7814. 10.1523/JNEUROSCI.0030-09.2009 19535592 PMC2840263

[B47] Nahir B. Jahr C. E. (2013). Activation of extrasynaptic NMDARs at individual parallel fiber-molecular layer interneuron synapses in cerebellum. J. Neurosci. 33, 16323–16333. 10.1523/JNEUROSCI.1971-13.2013 24107963 PMC3792467

[B48] Palay S. L. Chan-Palay V. (1974). Cerebellar Cortex: Cytology and Organization. Berlin; Heidelberg; New York, NY: Springer.

[B49] Rancillac A. Crépel F. (2004). Synapses between parallel fibres and stellate cells express long-term changes in synaptic efficacy in rat cerebellum. J. Physiol. 554, 707–720. 10.1113/jphysiol.2003.055871 14617674 PMC1664787

[B50] Satake S. Inoue T. Imoto K. (2012). Paired-pulse facilitation of multivesicular release and intersynaptic spillover of glutamate at rat cerebellar granule cell-interneurone synapses. J. Physiol. 590, 5653–5675. 10.1113/jphysiol.2012.234070 22930264 PMC3528983

[B51] Shouval H. Z. Bear M. F. Cooper L. N. (2002). A unified model of NMDA receptor-dependent bidirectional synaptic plasticity. Proc. Natl. Acad. Sci. U.S.A. 99, 10831–10836. 10.1073/pnas.152343099 12136127 PMC125058

[B52] Smith S. L. Otis T. S. (2005). Pattern-dependent, simultaneous plasticity differentially transforms the input-output relationship of a feedforward circuit. Proc. Natl. Acad. Sci. U.S.A. 102, 14901–14906. 10.1073/pnas.0505028102 16199519 PMC1253560

[B53] Soler-Llavina G. J. Sabatini B. L. (2006). Synapse-specific plasticity and compartmentalized signaling in cerebellar stellate cells. Nat. Neurosci. 9, 798–806. 10.1038/nn1698 16680164

[B54] Sun L. June Liu S. (2007). Activation of extrasynaptic NMDA receptors induces a PKC-dependent switch in AMPA receptor subtypes in mouse cerebellar stellate cells. J. Physiol. 583, 537–553. 10.1113/jphysiol.2007.136788 17584840 PMC2277014

[B55] Szapiro G. Barbour B. (2007). Multiple climbing fibers signal to molecular layer interneurons exclusively via glutamate spillover. Nat. Neurosci. 10, 735–742. 10.1038/nn1907 17515900

[B56] Turrigiano G. (2012). Homeostatic synaptic plasticity: local and global mechanisms for stabilizing neuronal function. Cold Spring Harb. Perspect. Biol. 4:a005736. 10.1101/cshperspect.a005736 22086977 PMC3249629

[B57] van Beugen B. J. Gao Z. Boele H. J. Hoebeek F. De Zeeuw C. I. (2013). High frequency burst firing of granule cells ensures transmission at the parallel fiber to purkinje cell synapse at the cost of temporal coding. Front. Neural Circuits 7:95. 10.3389/fncir.2013.00095 23734102 PMC3659283

[B58] Wulff P. Schonewille M. Renzi M. Viltono L. Sassoè-Pognetto M. Badura A.. (2009). Synaptic inhibition of Purkinje cells mediates consolidation of vestibulo-cerebellar motor learning. Nat. Neurosci. 12, 1042–1049. 10.1038/nn.2348 19578381 PMC2718327

[B59] Yamazaki T. Nagao S. Lennon W. Tanaka S. (2015). Modeling memory consolidation during post-training periods in cerebellovestibular learning. Proc. Natl. Acad. Sci. U.S.A. 112, 3541–3546. 10.1073/pnas.1413798112 25737547 PMC4371920

[B60] Yeung L. C. Shouval H. Z. Blais B. S. Cooper L. N. (2004). Synaptic homeostasis and input selectivity follow from a calcium-dependent plasticity model. Proc. Natl. Acad. Sci. U.S.A. 101, 14943–14948. 10.1073/pnas.0405555101 15466713 PMC522010

