# Supplementary material for: A Model of In vitro Plasticity at the Parallel Fiber—Molecular Layer Interneuron Synapses
Source: Front Comput Neurosci. 2015 Dec 24;9:150. doi: 10.3389/fncom.2015.00150 (PMC4689869; doi:10.3389/fncom.2015.00150)
Supplement: Supplementary file 1 [file DataSheet1.DOCX]

***Supplementary Material***

**A Model of Learning at the Parallel Fiber - Molecular Layer Interneuron Synapses**

**William Lennon*^1^, Tadashi Yamazaki^2^ and Robert Hecht-Nielsen^1^**

*** Correspondence:** William Lennon, Department of Electrical and Computer Engineering, University of California, San Diego, 9500 Gilman Drive, La Jolla, CA, 92093, USA.
wlennon@ucsd.edu

1. **Supplementary Figures**


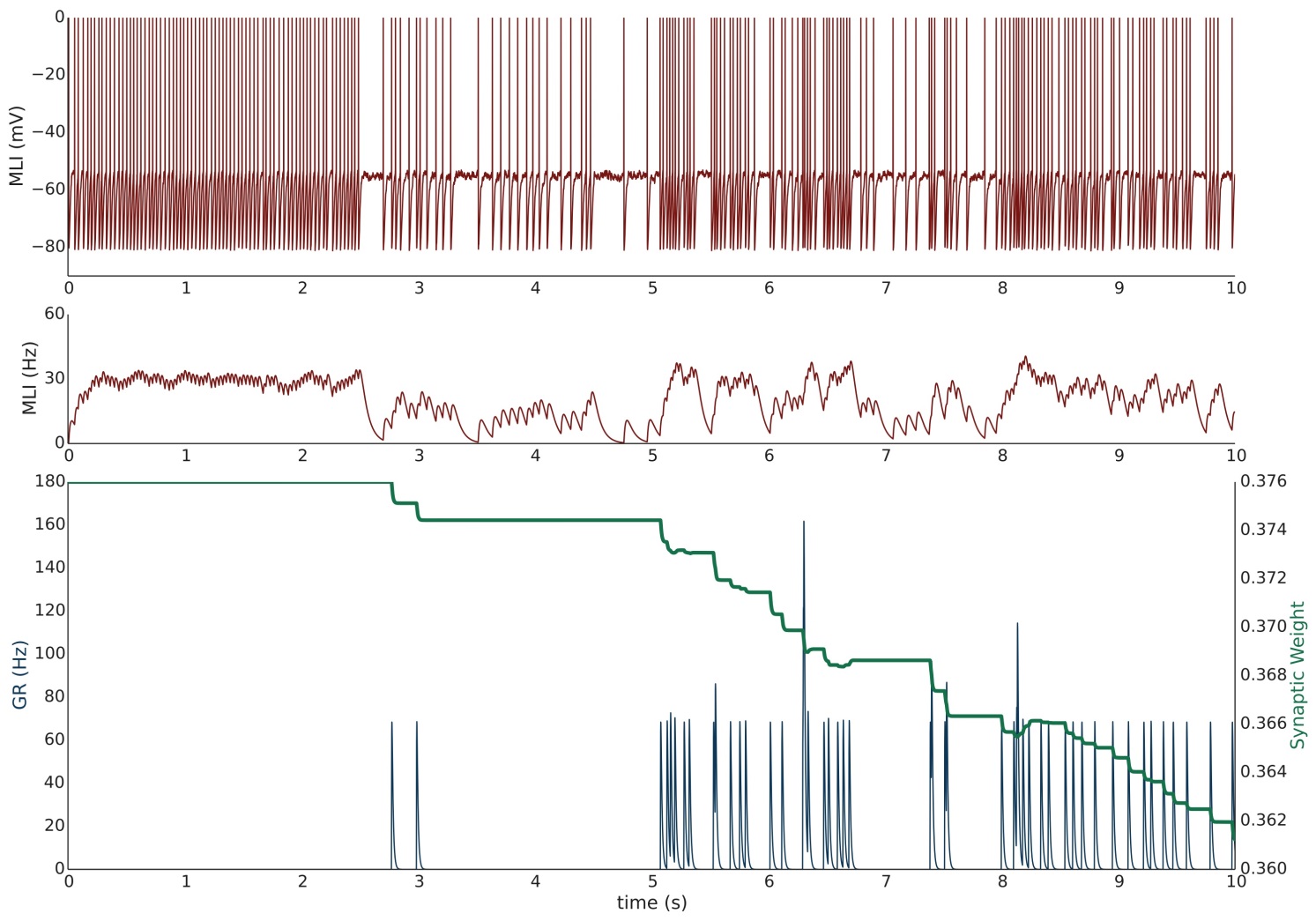


**Supplementary Figure 1. Simulation II.** Conventions similar to Fig. 1. See text for description.


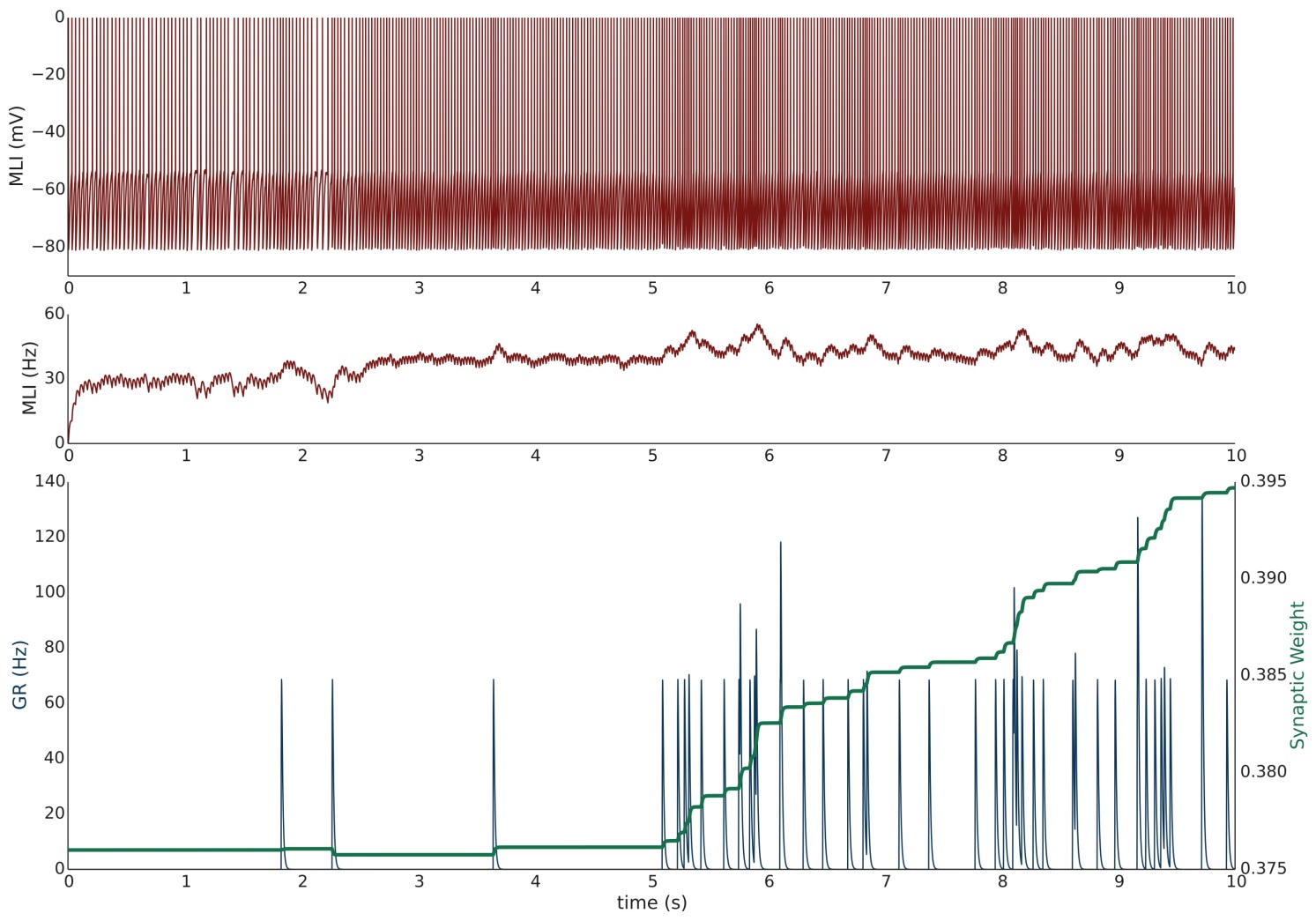


**Supplementary Figure 2. Simulation III.** Conventions similar to Fig. 1. See text for description.

**
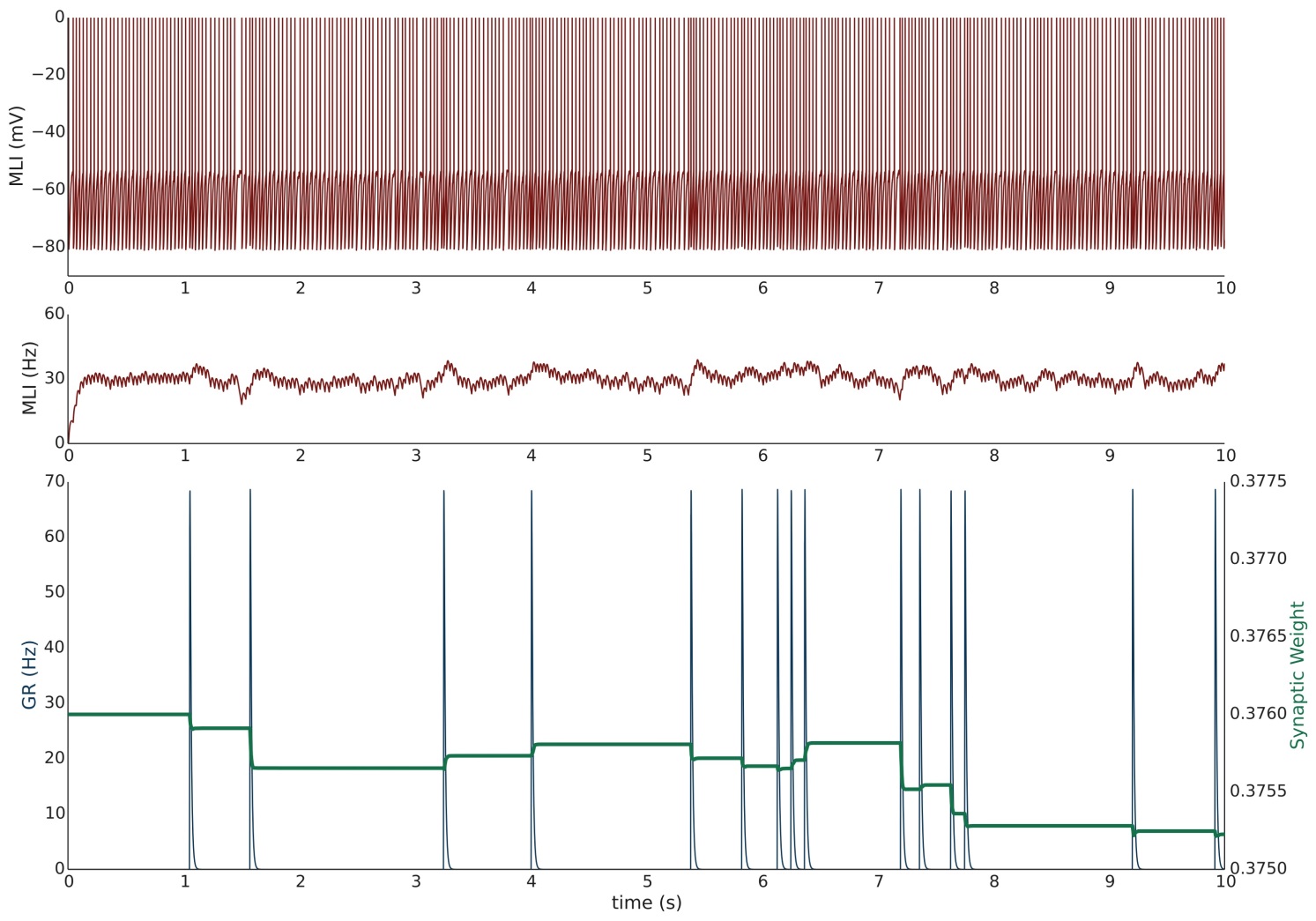
**

**Supplementary Figure 3. Simulation IV.** Conventions similar to Fig. 1. See text for description.

**
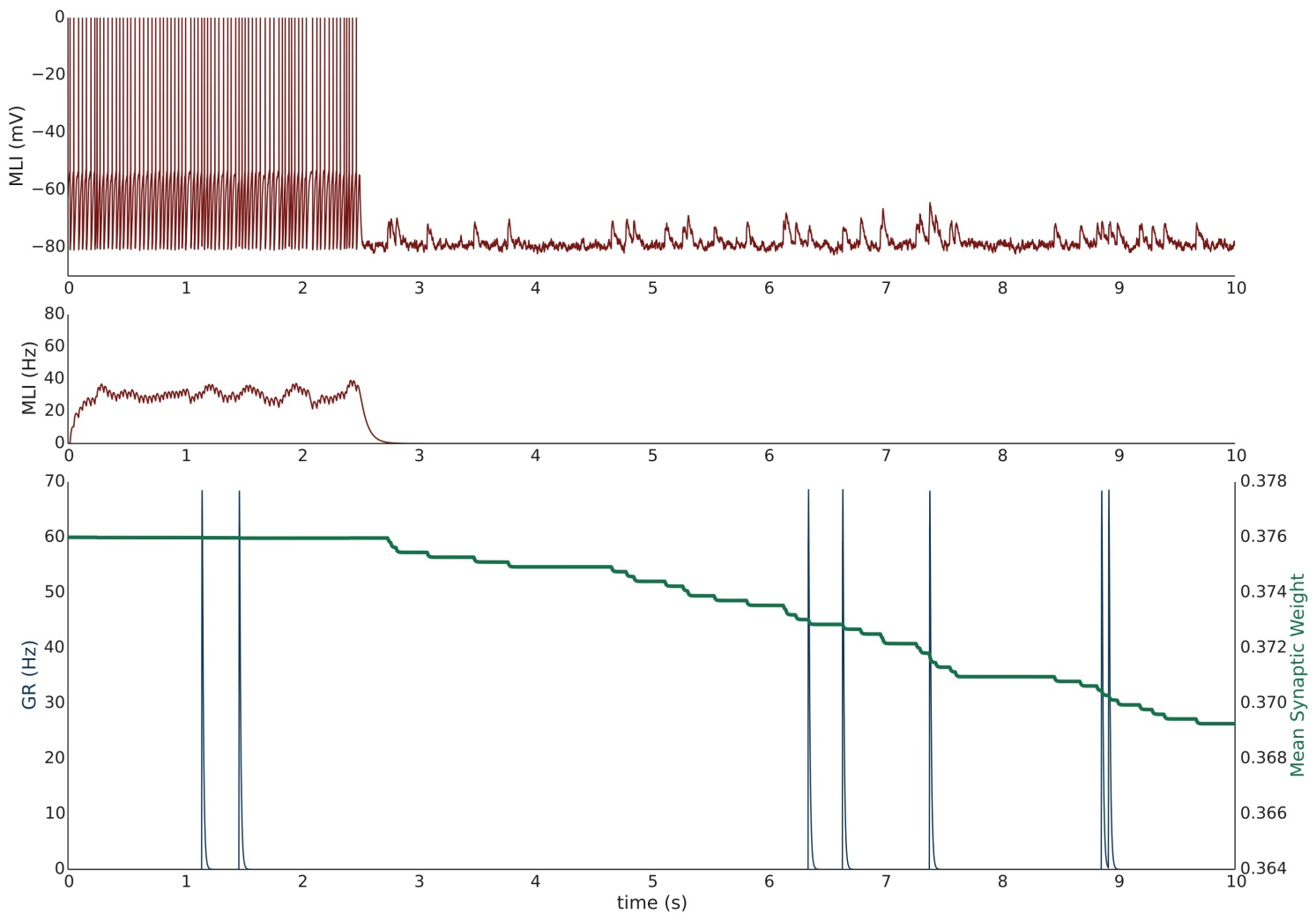
**

**Supplementary Figure 4. Simulation VII.** Conventions similar to Fig. 3. See text for description.

**
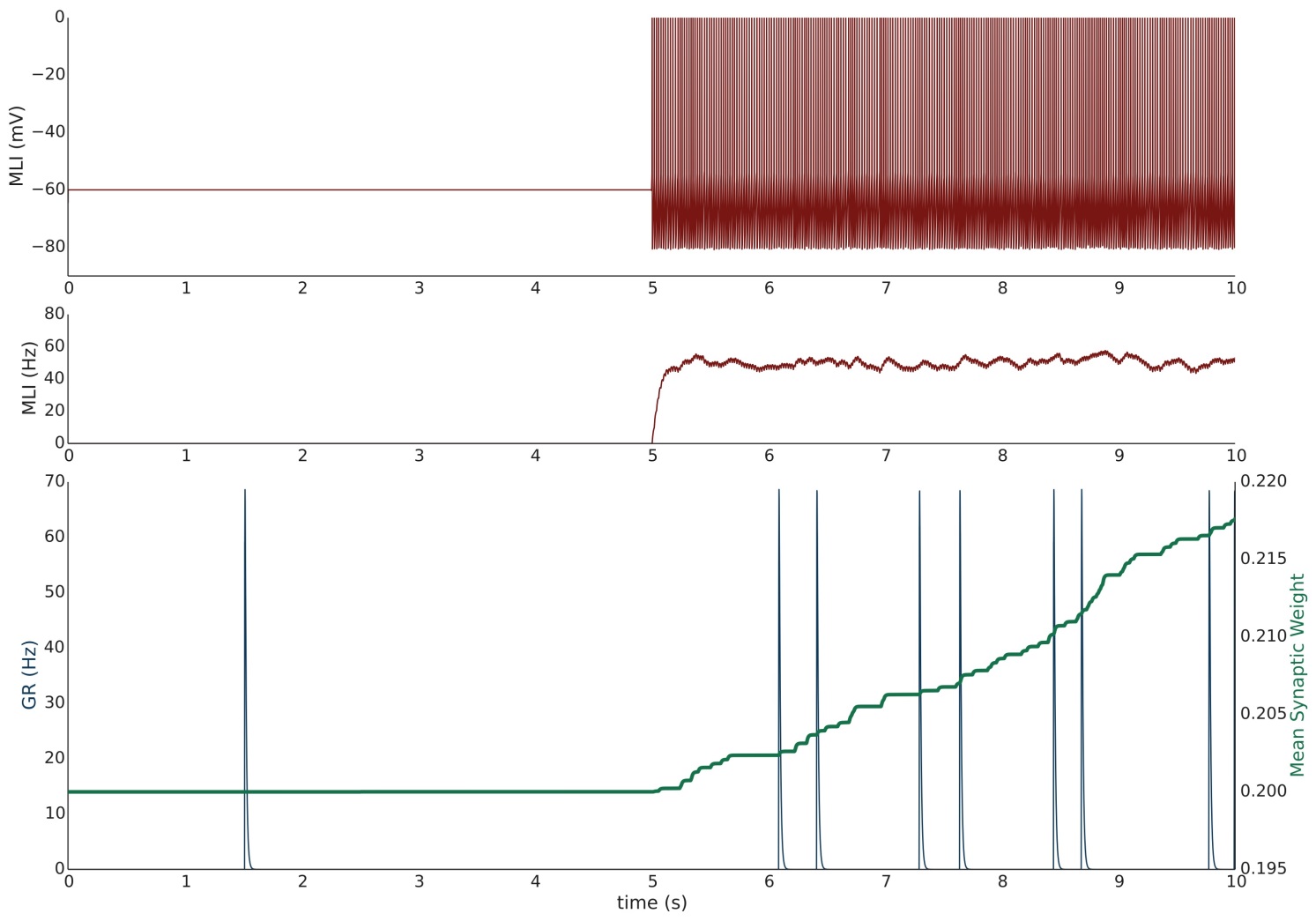
**

**Supplementary Figure 5. Simulation VIII.** Conventions similar to Fig. 3. See text for description.

**
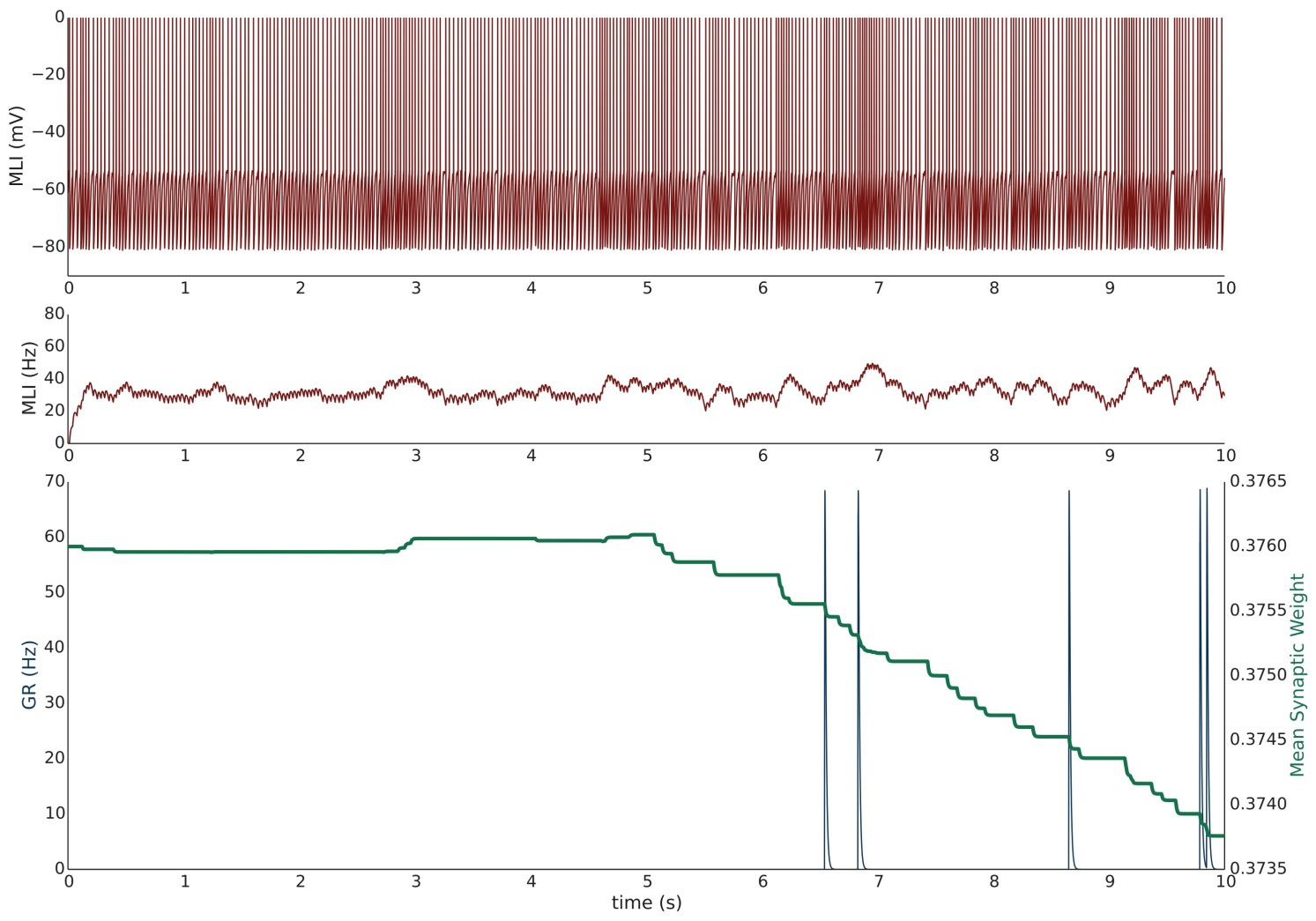
**

**Supplementary Figure 6. Simulation IX.** Conventions similar to Fig. 3. See text for description.

**
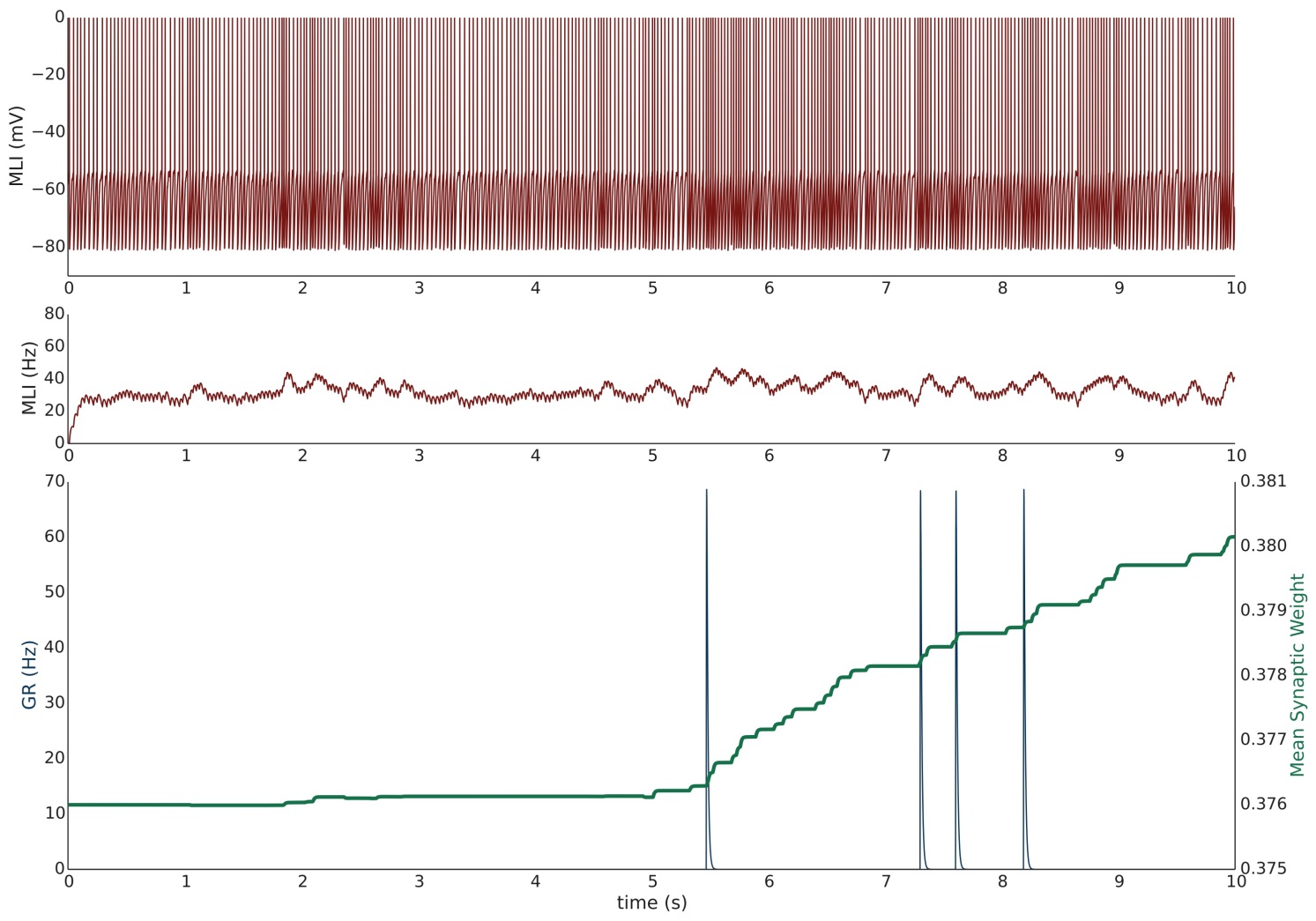
**

**Supplementary Figure 7. Simulation X.** Conventions similar to Fig. 3. See text for description.


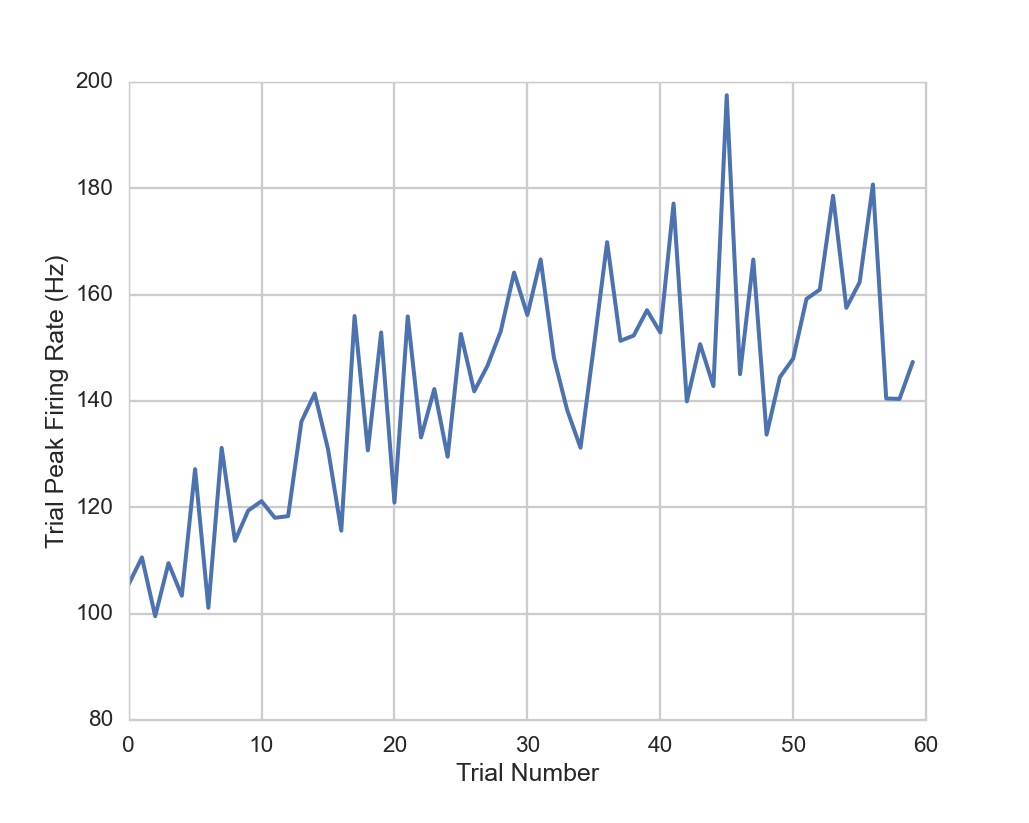


**Supplementary Figure 8. Peak firing rates by trial for Simulation VI.** This figure shows that the peak firing rates increase as the synaptic weight increases for Simulation VI.
